# Local habitual movement as a mechanism for Schistosoma mansoni transmission resurgence: a causal analysis of cross-sectional survey data

**DOI:** 10.1136/bmjph-2025-004500

**Published:** 2026-08-14

**Authors:** Rivka May Lim, Moses Arinaitwe, Simon A Babayan, Andrina Nankasi, Alon Atuhaire, Annet Namukuta, Mwima Nicholas, Amy B Pedersen, Joanne P Webster, Poppy H L Lamberton, Jessica Clark

**Affiliations:** 1 Institute of Evolution and Ecology, The University of Edinburgh, Edinburgh, UK; 2 School of Biodiversity, One Health and Veterinary Medicine, University of Glasgow, Glasgow, UK; 3 Vector Borne and Neglected Tropical Diseases Control Division, Republic of Uganda Ministry of Health, Kampala, Uganda; 4 Department of Pathobiology and Population Sciences, Royal Veterinary College, London, UK

**Keywords:** Disease Transmission, Infectious, Causality, Communicable Disease Control

## Abstract

**Background/aims:**

The WHO aims to eliminate schistosomiasis as a public health problem (EPHP) across 78 endemic countries, with interruption of transmission (IoT) as the goal in selected African countries by 2030. However, for low-prevalence settings that reach EPHP, guidance on managing transmission to maintain EPHP or move towards IoT is limited, partly due to insufficient knowledge of the drivers of persistence and/or re-emergence. In Uganda, some communities inland from Lake Victoria have achieved EPHP for *Schistosoma mansoni* but not progressed to IoT. This study aimed to assess whether routine, short-range travel to the highly endemic lake could sustain transmission in these settings.

**Methods:**

We conducted a cross-sectional study in five Ugandan villages ~5 km from Lake Victoria. Parasitological data were collected using Kato-Katz and Point-of-Care Circulating Cathodic Antigen tests alongside questionnaires on lake travel from 585 individuals aged 1–91 years. A structural causal model estimated the total and direct effects of travel frequency, activity type, water contact duration and drug treatment history on infection. Bayesian regression models and counterfactual simulations predicted infection under hypothetical interventions.

**Results:**

Reaching IoT in low-risk villages may be undermined by habitual short-range travel to high-risk sites driven by the nature and duration of lake contact. Daily lake-related travel caused a 1.7-fold increase in the odds of infection, while occupational activities caused a 3.4-fold increase compared with no lake activity. Counterfactual analysis showed that removing the lake contact duration variable most reduced infection risk among moderate-frequency travellers, while daily travellers showed smaller changes, and some transmission persisted among individuals with little or no lake contact. Simulations demonstrated that modifying lake contact behaviours could reduce individual infection risk but had limited population-level impact.

**Conclusion:**

These findings indicate that targeting only high-risk villages or individual behaviours is unlikely to achieve sustained, widespread IoT, underscoring the need for integrated control strategies that account for context-specific behaviours and local transmission ecology.

**WHAT IS ALREADY KNOWN ON THIS TOPIC**

- There is considerable guidance provided by the WHO for achieving elimination as a public health problem (EPHP) and interruption of transmission, based on a wealth of scientific evidence. However, evidence to support the development of resources for areas that have reached EPHP is limited, particularly with regards to when and how to scale back mass drug administration (MDA), while mitigating the risk of resurgence.
- Simulation studies suggest that reducing MDA with praziquantel in low-prevalence settings may trigger a return to higher transmission, but the mechanisms sustaining residual infections in these contexts remain unclear and are often identified through statistical associations rather than through robust causal analyses. As such, there is a lack of evidence identifying drivers of resurgence.
- Human mobility is recognised as a potential driver of schistosomiasis transmission. However, previous studies have focused on long-distance migration or spatial risk modelling to predict future impacts of movement. The impact of individual-level frequent, habitual, local travel between low- and high-prevalence areas on transmission and resurgence has received comparatively less attention.

**WHAT THIS STUDY ADDS**

- This study improved previous statistical associations by providing causal evidence that daily travel from low-prevalence inland villages to high-prevalence lakeshore landing sites increases the probability of *Schistosoma mansoni* infection risk and intensity, largely through activity type and duration of water contact.
- Counterfactual and mechanistic simulations showed that reducing lake-related travel would lower infection risk, but transmission would persist among individuals with little or no lake contact, suggesting that without MDA, travellers may sustain infection in their home villages.

**HOW THIS STUDY MIGHT AFFECT RESEARCH, PRACTICE OR POLICY**

- Our findings suggest that low-prevalence communities should not automatically receive less frequent MDA.
- Equally, nor should communities who have achieved EPHP see their MDA frequency reduced without understanding sources of and plans for mitigating re-introduction of infection.
- Surveillance in such settings should attempt to capture mobile individuals and leverage the sensitivity of antigen-based diagnostics to avoid underestimating residual risk.
- That communities are not isolated in space should be recognised when developing policy guidance, due to the risk of infection resurgence.
- Research and policy should continue to leverage causal approaches to clarify the mechanisms sustaining transmission in low-prevalence communities.

## Introduction

 The trematode *Schistosoma mansoni* causes the parasitic disease intestinal schistosomiasis. Transmission occurs through contact with freshwater that has been contaminated through open defecation or inadequate containment of stool infected with parasite eggs. Due to the localised range of the intermediate snail host, *S. mansoni* transmission is highly focal, with infection prevalence and intensity varying substantially even between neighbouring communities.^1^ In response to the significant health burden posed by schistosomiasis, the WHO recommends a coordinated set of interventions, primarily including mass drug administration (MDA) with praziquantel, in combination with health education, improved access to safe water and sanitation, and snail control.^2,3^ While these strategies have reduced prevalence in many settings, it remains unclear whether, when and how best to scale back MDA, with the 2021–2030 Neglected Tropical Disease Roadmap, developed by the WHO, highlighting the need to understand how to monitor for and identify mechanisms that could drive resurgence.^4^

The WHO outlines three sequential programmatic goals to guide control efforts against schistosomiasis: (1) morbidity control; (2) elimination as a public health problem (EPHP) and (3) interruption of transmission (IoT).^2^ A community is considered to have achieved morbidity control when <5% of school-aged children (SAC) are heavily infected (defined as ≥400 eggs per gram of faeces (EPG) for intestinal schistosomiasis), EPHP is achieved when <1% of SAC are heavily infected and IoT is defined as having no autochthonous human or snail cases for five consecutive years.^2^ While the WHO provides clear guidance towards achieving EPHP in moderate and high prevalence settings,^2^ there are no defined indicators for when to stop MDA or how to carry out surveillance. Simulation models have indicated that when prevalence is <1% by faecal egg counts, residual persistent transmission can lead to resurgence without sufficient treatment^5^ and surveillance.^6^ The impact of this is evidenced by resurgence in China following the reduction of interventions^7^ and in Zanzibar after a 1-year pause in MDA.^8^ Without clear guidance on how to detect early signs of resurgence or respond effectively, areas approaching EPHP or IoT risk losing any progress made. To mitigate this risk, it is essential to identify the mechanisms that sustain low-level transmission and may drive resurgence, to inform surveillance and treatment strategies critical to long-term elimination and interruption success.

Communities living on the shores of Lake Victoria, Uganda tend to rely heavily on the lake for daily life. Despite two decades of MDA, many continue to experience high rates of *S. mansoni* transmission.^9,10^ In contrast, some inland communities have shown reductions in prevalence, with some reaching EPHP, although without achieving IoT.^11^ This raises the question of whether inland communities which have reached EPHP then fail to progress to IoT is due to their proximity to high-endemicity regions, and specifically whether transmission is sustained through habitual human movement between these low- and high-risk locations.

Local habitual human mobility between low and high transmission regions has received comparably less attention than long-term/distance human migration or movement.^12–15^ Where previous studies have identified human mobility as a key factor shaping the spatial dynamics of schistosomiasis transmission, they have largely relied on indirect evidence, such as spatial models of risk or ecological correlations, to infer associations between movement and infection^16–18^ and have not focused on the impact of habitual movement between areas which have achieved EPHP and those where endemicity remains high. Existing control strategies often operate by administrative units, assuming that populations are geographically isolated, and as such likely obscuring the possibility that habitual local mobility could mean individuals act as a conduit for sustained transmission. This raises another important question of whether certain people should or can be prioritised for surveillance or treatment during the elimination phase, as a function of their connectivity to high-risk locations, to prevent re-establishment of local transmission.

Additionally, although travel-related behaviours such as water contact and time spent in the lake are known risk factors for schistosomiasis transmission,^19^ there is little evidence on whether particular individuals, or their activities are the cause of this risk. Understanding whether routine travel directly increases infection risk, or whether it acts indirectly through other behaviours is essential to inform targeted elimination strategies. Traditional epidemiological analyses often describe statistically significant associations between exposures and outcomes but cannot determine whether those exposures drive the outcomes (ie, evidence causality). In contrast, causal inference methods, like structural causal modelling (SCM), are specifically used to estimate the effects of exposures while accounting for confounding, mediation (where effects operate through intermediate variables) and complex interdependencies. These approaches are increasingly applied in infectious disease epidemiology to generate policy-relevant insights, where programmatic decisions must be made using observational data.^20,21^ By explicitly defining and testing assumptions regarding the ways different drivers might interact, and the pathways through which transmission is shaped, SCMs offer a rigorous framework to evaluate drivers of infection and support elimination planning.

The aim of this study was therefore to quantify the causal effect of habitual, local travel to and from highly endemic Lake Victoria areas on *S. mansoni* infection risk in regions that have met WHO targets for EPHP. To do this, we applied a Bayesian causal inference modelling framework to highly detailed behavioural and parasitological data collected from five communities to estimate the direct and indirect effects of travel-related behaviours on infection risk. Understanding these patterns of movement and their relationship to infection can offer valuable insight into whether such individuals may be contributing to ongoing transmission that could be hindering progress towards IoT. Clarifying this relationship will support the development of efficient and effective surveillance strategies, improve disease resurgence detection and ultimately contribute to achieving IoT goals.

## Methods

### Patient and public involvement

Community members enrolled voluntarily and were first engaged during a sensitisation visit led by the Ministry of Health, Uganda (MoHU) team. While the public did not directly inform study design, outcome measures or recruitment; participation was voluntary following the sensitisation meetings, during which questions were addressed before consent. Feasibility was thus dependent on community acceptance. The pilot questionnaire was refined based on volunteer feedback.

The research questions were developed from WHO and MoHU policy priorities to understand community experiences and practices related to schistosomiasis and its treatment, particularly in low-prevalence areas where data are limited. Feedback at the community level was provided after a post-treatment follow-up 12 months after these data were collected.

### Data collection

**Figure 1:**
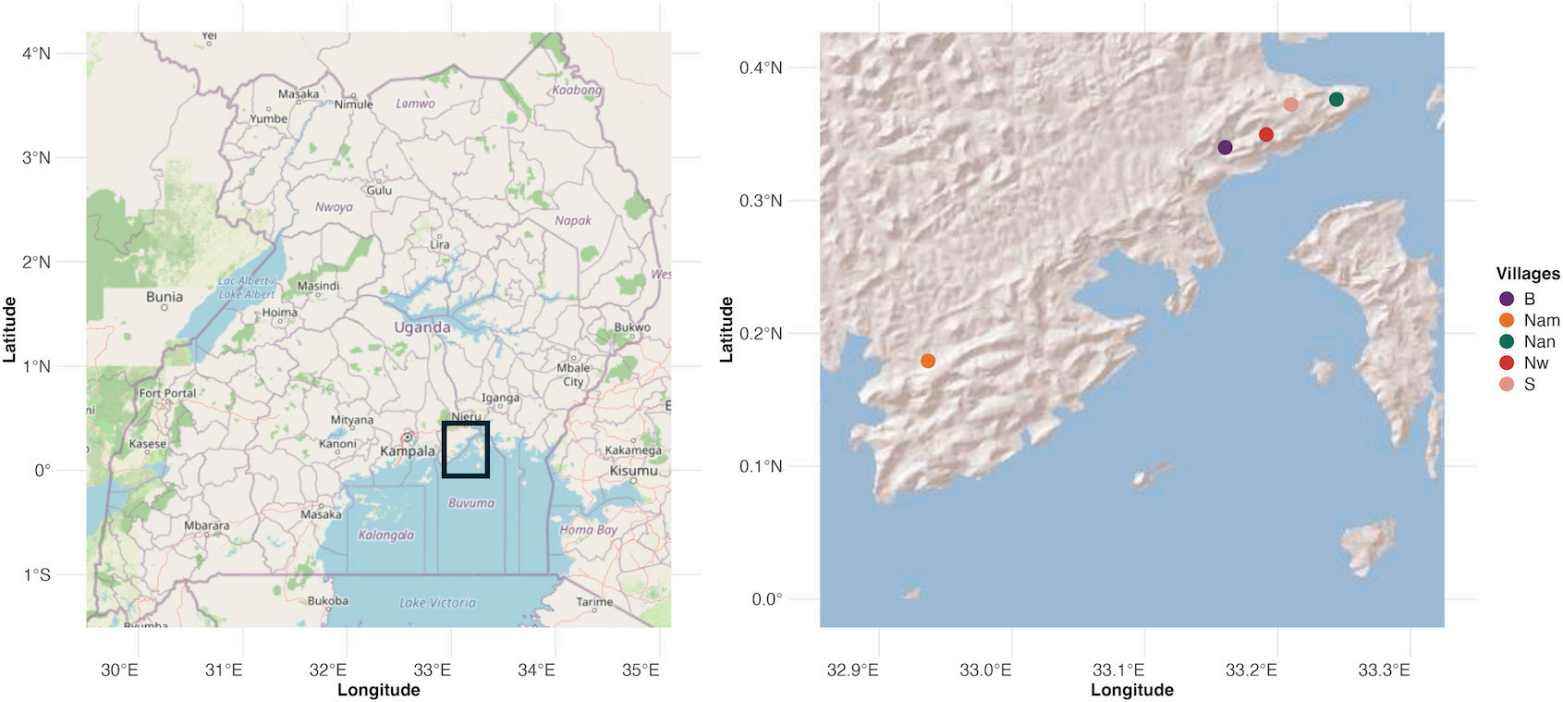
Map of the study area showing the locations of the five participating villages along the northern shore of Lake Victoria, Uganda. Each point represents one village, colour-coded by name: B (purple), Nw (red), Nam (orange), Nan A (green) and S (pink). The right panel displays approximate locations of each village, while the left panel provides a broader map of Uganda, with a black rectangle indicating the location of the zoomed-in map.

Five villages each ~5 km from Lake Victoria, Uganda were selected: B, Nam, Nan, Nw and S (figure 1) (see online supplemental data ‘Village selection’ for full selection protocol). Individuals aged 1–91 years were recruited, assisted by the village health team. The study took place in each village for 1 week, from October 2021 to December 2021.

Three stool samples were collected per person over 3 days and duplicate Kato-Katz examined by experienced technicians, under a microscope, for *S. mansoni* eggs.^22^ One urine sample per person was tested using Point-of-Care Circulating Cathodic Antigen tests (POC-CCAs), using the 1–10 G-Score system.^23^ Kato-Katz has 100% specificity but POC-CCAs were included to improve sensitivity in low prevalence settings.^24^ A participant was considered positive for *S. mansoni* if at least one egg was observed in a stool sample, or if negative by eggs but a POC-CCA ≥G3.^25,26^ Prevalence was calculated by dividing the number of positive people by the full sample size. Individual mean infection intensity (EPG) was calculated as the mean slide count multiplied by 24. Population-level mean EPG was calculated by removing individuals with EPG=0.

Human mobility and water contact were assessed through a questionnaire administered once during the study period. Questionnaires were conducted in the local language and/or dialect by trained fieldworkers and answers recorded on tablets with KoboToolBox (see online supplemental table S1 for the full questionnaire). Respondents were asked their age, sex, information on current residency, questions related to lake interactions including how many times they had travelled to Lake Victoria in the past 3 months ((a) never, (b) just once, (c) once a month, (d) two times a month, (e) once a week, (f) two times a week or (g) daily), what activities they engaged in on arrival, how long they spent in the water, and MDA participation. Age was categorised into three programmatically relevant groups: preschool-aged children (PSAC) (0–5 years), SAC (6–15 years) and adults (>15 years). We explored an alternative four-level specification including young adults (15–25 years) in sensitivity analyses. As effect estimates for young adults were comparable to those for older adults, with no improvement in model fit or predictive accuracy, the three-level age variable was retained for the analysis. Participants reported a wide variety of activities undertaken at the lake, which were grouped into (a) domestic, (b) occupational, (c) recreational and (d) trade/visit. A complete list of activity responses and their groupings is provided in online supplemental table S2.

### Directed acyclic graph construction

**Figure 2:**
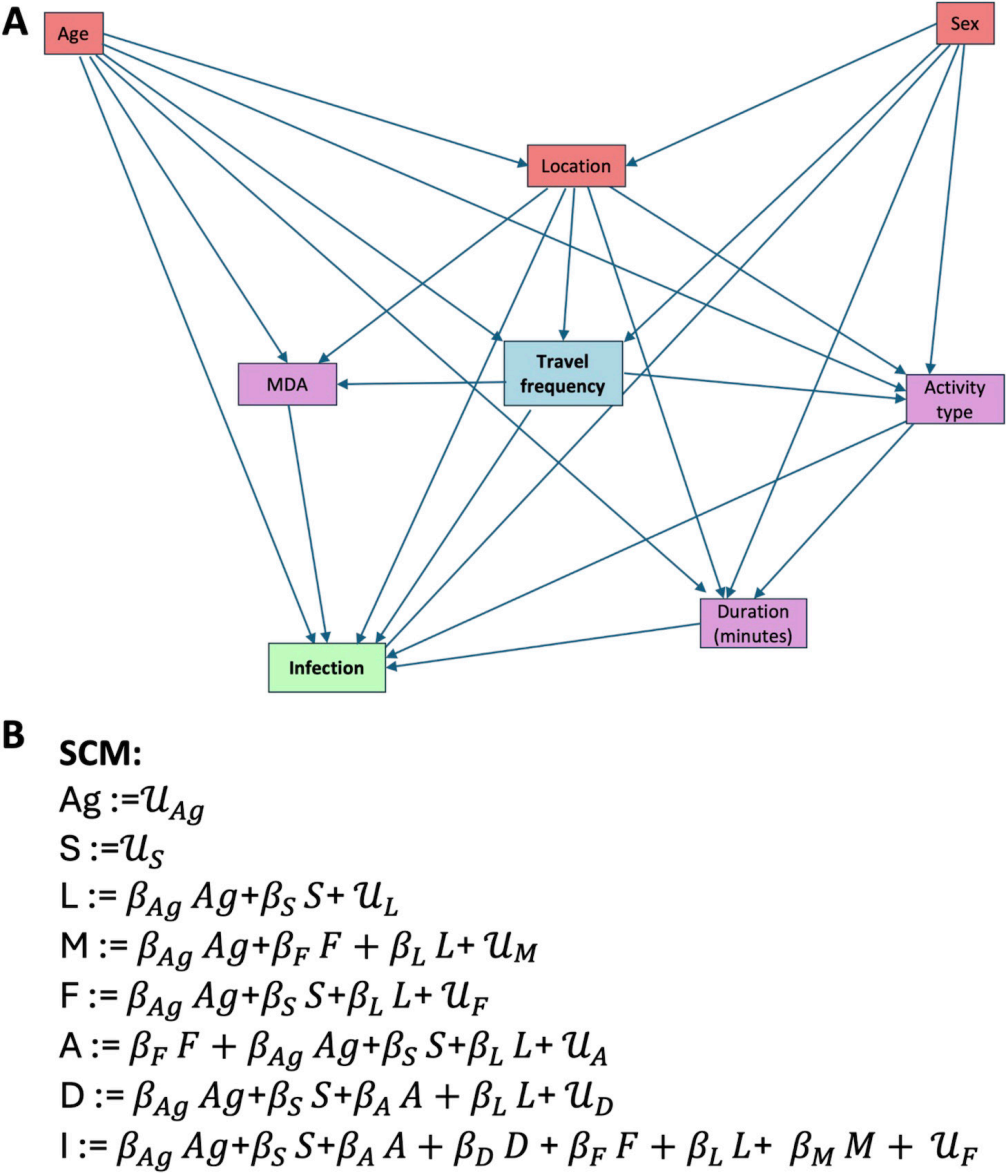
Directed acyclic graph (DAG) representing hypothesised causal relationships between variables and *Schistosoma mansoni* infection (**A**), with structural equations for each node (**B**). (**A**) The DAG outlines the assumed causal structure linking demographic, behavioural and environmental variables to infection. Arrows represent direct causal effects. Key exposure variables include travel frequency, activity type and duration of water contact, with potential confounders such as age class, sex and location. (**B**) Structural equations define the dependencies of each variable, with terms representing regression coefficients (β), unobserved residual error (𝒰) and observed covariates. A, activity; Ag, age class; D, duration in the lake; F, frequency of travel; I, infection; L, location; M, mass drug administration (MDA); S, sex; SCM, structural causal modelling.

All analyses and visualisations were executed using R (V.4.4.3). A directed acyclic graph (DAG) was constructed to represent hypothesised causal relationships influencing the link between an individual’s infection status and travel to a highly endemic location. The DAG was then used to inform the structure of the final model used for analysis. This DAG included travel frequency to Lake Victoria, water contact duration at Lake Victoria, activity type at the lake, MDA history and demographic covariates: age, sex and village of residence (figure 2A). The structure of the DAG, that is, direction of the edges and which variables interact, was informed by a combination of domain knowledge, published literature and expert opinion of schistosomiasis transmission dynamics, local environmental conditions and behavioural patterns.^27^ The DAG aimed to visually represent a biologically plausible and theoretically grounded causal framework. The DAG was visualised using the R package dagitty.^28^ The structure of the DAG was validated against the observed data by testing the implied conditional independencies between the variables (see online supplemental data ‘Model validation and restructuring’ for a full explanation and DAG structure and counterfactual simulation sensitivity analysis for further validation of structure).

### Structural causal model

From the fully validated DAG, an SCM was constructed for analysis. Each variable was expressed as a function of its direct causes (parents in the DAG) and an independent noise term (figure 2B). These functions define the joint distribution of all variables in the system, informing the underlying causal structure. Minimal adjustment sets for each exposure-outcome relationship were estimated from the SCM to ensure an unbiased estimation of total and direct effects (online supplemental table S3).^29,30^ These adjustment sets were formulated using causal graph theory principles based on chains, forks and colliders.^31^ To account for uncertainty and provide probabilistic estimates of causal effects, we used Bayesian linear regression models. Each edge in the DAG was parameterised using these Bayesian models. Effects were considered supported if the 95% credible interval (95% crI) for the effect between variables did not include zero after adjusting for the conditioning set identified by the DAG structure. Using these appropriate adjustment sets, we estimated total and direct effects of key causes of infection status and intensity, fitting models for each of the four primary causes: travel frequency, water contact duration, MDA history and activity type. Bayesian linear models were fitted, and posterior predictive checks conducted using the *brms* package in R.^32^ Binary infection status (*S. mansoni* presence or absence) was modelled using a Bernoulli distribution with a logit link. Egg burden (measured as mean EPG, among egg-positive individuals only) was modelled using a Gamma distribution with a log link, given its positive and right-skewed distribution. After carrying out sensitivity analyses for choice of coefficient priors (online supplemental data ‘Sensitivity analysis of priors for the coefficients’ and online supplemental figure S1), weakly informative priors were chosen and differed by model family. Priors for each of the models are shown in supplementary data (online supplemental table S4). Posterior distributions were drawn using four chains of 4000–10 000 iterations each (typically with 30% warm-up), depending on model complexity. MCMC convergence was assessed using R-hat statistics and effective sample sizes (online supplemental table S5).

### Counterfactual interventions

To simulate counterfactual interventions, we used posterior draws from the total effect models to generate predicted probabilities of infection and predicted mean EPG under both observed and intervened exposure conditions. For each intervention, we selected a target exposure variable (travel frequency, water contact duration and activity type) and modified it to reflect the desired intervention. Where an exposure had downstream effects on other variables in the DAG (figure 2A), these variables were also modified; for example, if someone does not travel to the lake, duration in the lake would also be set to zero.

The original model, fitted with the unmodified factual dataset using the appropriate adjustment sets (online supplemental table S3), was then applied to both the factual and counterfactual datasets to obtain posterior predictions. This preserved the natural variation in covariates needed to estimate P(Y|X) when applying the *do*-calculus, where Y denotes the outcome (binary infection status or mean EPG) and X the exposure of interest.

This approach enabled us to estimate how infection risk would change under hypothetical interventions while avoiding post-treatment bias. We applied this approach to different scenarios: (a) *Travel frequency*: setting all daily travellers to no travel, examining (1) change in infection probability for the entire population and (2) change only for daily travellers; (b) *Activity type*: (1) changing all ‘occupation’ activity to ‘none’ and examining infection probability for the whole population and occupational travellers only, (2) changing all ‘domestic’ activity to ‘none’ and examining mean EPG for the whole population and for domestic-activity individuals only; and (c) *Water contact duration*: setting duration to zero minutes, examining (1) change in infection probability for the entire population and (2) change only for those who had reported lake contact.

For each scenario, posterior predictive distributions were summarised as means across individuals for each posterior draw and visualised under observed and counterfactual conditions using histograms. Presenting full distributions rather than single point estimates allowed assessment of uncertainty, skew and variability in predicted effects.

### Mechanistic counterfactual analysis

To examine whether the effect of removing lake contact duration varied by travel frequency, we conducted a mechanistic counterfactual analysis. Posterior infection risks were predicted under the observed data and under a counterfactual scenario where duration was set to zero for individuals who had reported >0 mins in the Lake. Conditional average treatment effects were then estimated by computing mean infection risks within each travel-frequency group and taking the difference between counterfactual and observed values. This approach quantified how the causal effect of removing lake contact duration differed across levels of travel frequency, providing a sensitivity analysis of heterogeneity in the duration-infection pathway.

### DAG structure and counterfactual simulation sensitivity analysis

To quantify the impact of potentially unmeasured confounding variables on our findings, we calculated E-values following the methods of.^33^ The E-value represents the minimum strength of association that an unmeasured confounder would need to have with both the exposure and the outcome simultaneously to fully explain the observed association. For binary outcomes, odds ratios were converted to risk ratios using the observed baseline infection risk in the reference group. For burden models (Gamma, infected only), mean ratios were used. E-values were calculated for both the point estimate and the credible interval bound closest to null.

The water-contact-duration variable was most susceptible to self-reporting bias, because it is a continuous measure of time rather than a categorical variable, making precise recall inherently more difficult. Social desirability bias may also have caused underreporting because participants may have reduced the duration reported to give an answer they thought researchers wanted. To assess the validity of reported durations, we used granular individual-level activity data (prior to grouping into broader categoriesOnline supplemental file 1) and examined whether reported durations were consistent with the biological plausibility of each activity. For example, fishing could involve hours of water contact, whereas collecting water could not. To formally test the effect of under-reporting, we applied a proportional underreporting correction to the duration coefficient from the duration to infection model. If participants report only a fraction of their true water contact time, the observed coefficient underestimates the true effect by the same fraction. We applied this correction across reporting fractions 0.50–1.0 and reported corrected cumulative odds ratios at 30, 60 and 120 min of water contact.

In our DAG, we presented MDA participation, activity at the lake and duration in the lake as mediating variables between travel frequency and infection. However, each could act as confounding variables of this relationship. To test these structural assumptions, we compared estimates across all eight combinations of treating each mediator as a confounder under different scenarios: no conditioning (total effect, correct under our DAG), each mediator individually, all pairs and all three simultaneously. Models were compared for daily travel versus never travel.

Two assumptions in our counterfactual analysis may cause us to overestimate or underestimate the true population-level effect of the proposed interventions. First, our analysis attributed any reduction in cercarial contamination solely to the daily travellers who change their behaviour. However, waterways are shared resources; if daily travellers reduce their water contact, other individuals may also benefit from lower cercarial concentrations, even without changing their own behaviour. To explore how much this shared environmental benefit might amplify our estimates, we re-ran our analysis under four scenarios in which 0%, 10%, 20% or 30% of the contamination reduction achieved by daily travellers was assumed to also benefit non-daily-travellers. Second, our counterfactual analysis assumed that travellers who reduce contact with one waterway do not simply shift that contact to another waterway. In practice, some proportion may displace rather than reduce their water exposure. To bound this, we re-ran our analysis under scenarios in which 10%, 20%, 30%, 40% or 50% of daily travellers were assumed to relocate their water contact to a different waterway rather than reduce it altogether and modelled the effect on non-daily travellers.

## Results

### Prevalence of *S. mansoni* infections

Community-wide prevalence of infection was 51% in Nan, 45% in B, 33% in Nam, 30% in Nw and 27% in village S (mean=36%). No participant had an infection intensity of ≥400 EPG (online supplemental table S6). As the WHO defines EPHP as <1% of SAC with ≥400 EPG, all villages had achieved EPHP.^2^ For more detailed village, age and sex level variation stratified by travel habits (online supplemental tables S7 and S8 and online supplemental figure S2). Observed infection prevalence and intensity by behavioural and exposure variables can be visualised in online supplemental figure S3.

### Effect of travel frequency on infection

Among all participants, 56% (329/585) reported travelling to the lake at least once in the last 3 months (online supplemental figure S3). Of these travellers, 41% (134/329) were infected; in addition, 30% of those that did not travel to the lake were infected (77/256). Daily travel to the lake was reported by 24% of participants (138/585). The mean EPG in each group (travel and no travel) was not meaningfully different; 11.3 (SD=22.2) and 11.6 (SD=13.4), respectively.

**Figure 3:**
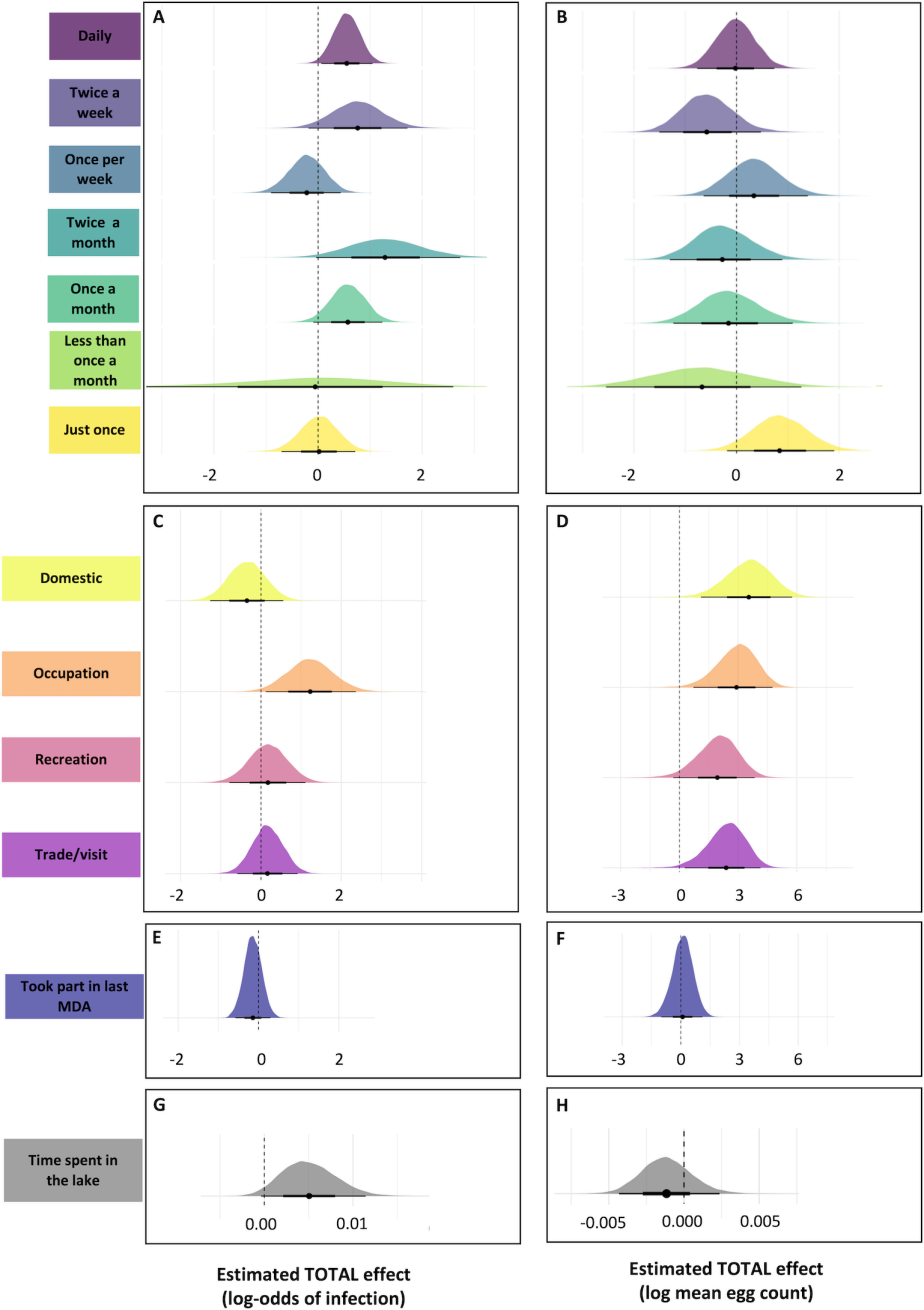
Estimated total effects of lake-related behaviours on *Schistosoma mansoni* infection and infection burden. Each pair of panels (left=infection risk; right=infection burden) shows posterior distributions from Bayesian regression models estimating the total effects of a specific lake-related variable. Self-reported travel frequency to Lake Victoria (relative to no travel) on (**A**) the probability of *S. mansoni* infection and (**B**) infection burden (measured as eggs per gram (EPG) of stool). Self-reported lake-related activity type on (**C**) infection probability and (**D**) infection burden. Participation in the most recent mass drug administration (MDA) on (**E**) infection probability and (**F**) infection burden. Time spent in contact with lake water on (**G**) infection probability and (**H**) infection burden. Densities represent marginal posterior distributions of log-odds (infection) or log-mean EPG (burden). Thick and thin horizontal lines represent 66% and 95% credible intervals, respectively. The vertical dashed line indicates the null effect (log-odds or log-mean=0). In (A), travel frequency categories are ordered from highest (daily visits) to lowest (single visit).

The total effect of travel frequency on infection was estimated using the minimal adjustment set of confounders only (online supplemental table S3). There was a positive total causal effect for those who travelled once a month, two times a month, two times a week and daily to Lake Victoria in the last 3 months, with daily visits having a significant 1.7× effect on increasing infection risk (log odds=0.55, 95% crI 0.06 to 1.05) (figure 3A). When estimating the direct effect, there was no longer a significant effect of daily visits (online supplemental figure S4), with the activities classed as occupation now having the only significant effect in the model. Using a separate model of EPG, the amount of travel undertaken did not significantly affect the EPG in either the total or direct models (figure 3B and online supplemental figure S5).

### Effect of activity carried out while visiting Lake Victoria

When estimating the total effect of activity on infection, individuals reporting occupational water contact had higher log-odds of being infected compared with those with no lake contact (log-odds=1.23; 95% crI 0.12 to 2.36). Other activities were not significant predictors of infection risk (figure 3C).

Including water contact duration in the model to estimate the direct effect reduced the estimated effect of occupational activity on infection (log odds=0.91; 95% crI –0.26 to 2.10), compared with the model without duration (online supplemental figure S6). Duration itself was positively associated with infection risk (log-odds=0.01, crI 0.0 to 0.01), confirming partial mediation as outlined in the DAG (figure 2A) and sensitivity analysis in online supplemental data.

All activities were associated with a higher mean EPG compared with those reporting no lake-related activity. Domestic activities had the greatest effect with a 35× higher mean EPG than those reporting no activity at the lake (log odds=3.55, 95% crI 1.10 to 5.76) (figure 3D). Adjusting for duration of lake exposure in a direct effect model, did not meaningfully alter the association between activity type and EPG in comparison to the total effect model (online supplemental figure S7), and duration was not a significant predictor.

### Effect of taking part in the last MDA

Across all villages, 73% (425/585) of participants said they took part in the last MDA. Details of responses to why a participant did not take part in the last MDA and what age groups did take part can be found in online supplemental tables S9 and S10. When modelling the effect of MDA participation on infection, the total and direct effects used the same adjustment sets as there were no recorded mediators (online supplemental table S3). Individuals who reported participating in MDA had lower though non-significant log-odds of infection than those who did not (log-odds=–0.14; 95% crI −0.57 to 0.29) (figure 3E). There was no association between MDA and mean EPG (log-mean=0.09; 95% crI –0.99 to 1.11) (figure 3F).

### Effect of time spent in Lake Victoria

The longest lake visits were for occupational activities (mean time spent=92 min, SD=161), recreation followed (mean=33, SD=32), then trade/visiting (mean=18, SD=34), with domestic activities shortest (mean=14, SD=32). For daily visits, occupations were rare (n=9) but longest (mean=64, SD=70), while domestic visits were most common (n=73) and shortest (mean=5, SD=12) (online supplemental table S11).

When modelling the effect of time spent in Lake Victoria on infection, the total and direct effects used the same adjustment sets as there were no mediators (online supplemental table S3). The log-odds of infection increased by 1% (log-odds=0.01, 95% crI −0.0 to 0.01) for every additional minute (figure 3G) (note that the lower bound is essentially on 0 suggesting an incalculably small possibility of 0, and so technically only a weekly positive association). While this effect is small per minute, it accumulates across longer durations, for example, an additional 60 min corresponds to approximately 82% higher odds of infection than someone who spends no time in the lake (assuming linearity) (log-odds of (0.01× 0.6)). There was no effect of duration on mean EPG (log-odd=−0.0, 95% crI −0.0 to 0.0) (figure 3H).

### Counterfactual interventions

**Figure 4:**
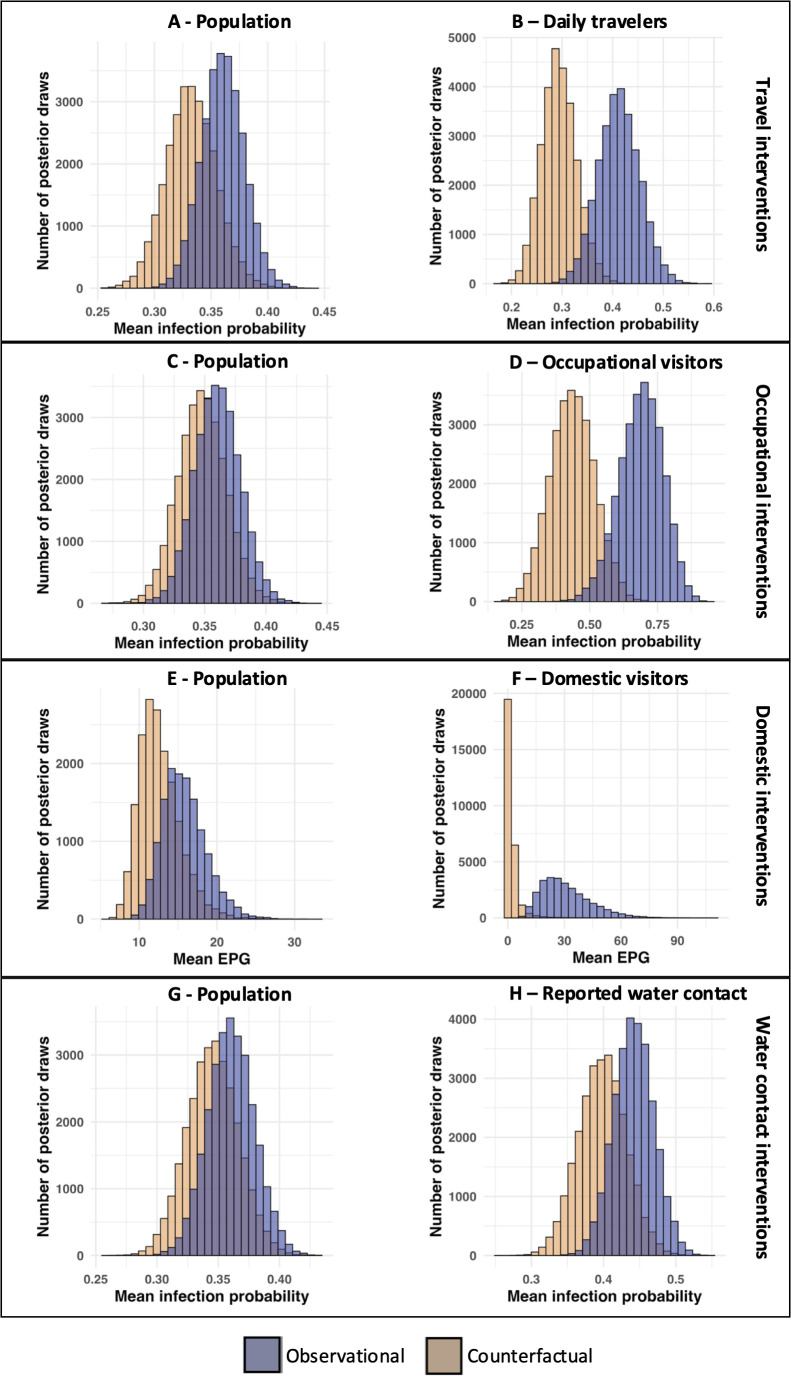
Counterfactual predictions of *Schistosoma mansoni* infection probability under reduced travel and water contact scenarios. Posterior distributions of predicted infection probabilities for selected subgroups under observed (blue) and counterfactual (orange) conditions, based on Bayesian logistic regression models. (**A and B**) Travel interventions: the predicted infection probability of no daily travel on the entire population (**A**); and only daily travellers (**B**). **(C and D**) Occupational interventions: the predicted infection probability of no one doing occupational activities on the entire population (**C**); and only those going for occupational tasks (**D**). **(E and F**) Domestic interventions: the predicted mean eggs per gram of no one doing domestic activities on the entire population (**E**); and only those going for domestic tasks (**D**). **(G and H**) Water contact interventions: the predicted infection probability of no water contact on the entire population (**G**); and only those who reported making contact (**H**). Distributions represent posterior samples of individual infection probabilities aggregated across the sample population.

The first counterfactual simulation removed daily lake travel, reducing infection probability by 2.8% in the whole population from 36% (95% crI 32% to 40%) to 33.2% (95% crI 29.0% to 37.6%) (figure 4A) and by 11.9% among daily travellers from 41.3% (95% crI 33.5% to 49.3%) to 29.2% (95% crI 23.0% to36.4%) (figure 4B). Removing occupational lake visits reduced infection probability by 1.2% in the whole population from 36.0% (95% crI 32.4% to 39.8%) to 34.8% (95% crI 31.0% to 38.7%) (figure 4C) by 25.5% among those reporting occupational visits, from 69.3% (95% crI 52.5% to 83.5%) to 43.5% (95% crI 27.8% to 59.4%) (figure 4D). Eliminating domestic water activities reduced mean infection intensity from 15.5 EPG (95% crI 11.1 to 22.22) to 12.2 EPG (95% crI 8.5 to 18.9) in the overall population (figure 4E) and from 28.6 EPG (95% crI 12.1 to 62.1) to 1.2 EPG (95% crI 0.2 to 11.0) among those reporting domestic activities (figure 4F).

Setting lake contact duration to zero reduced infection probability from 36.0% (95% crI 32.40% to 39.8%) to 34.5% (95% crI 30.5% to 38.6%) in the overall population (figure 4G) and from 44.2% (95% crI 38.7% to 49.7%) to 39.8% (95% crI 33.3% to 46.2%) among those reporting contact (figure 4H), a reduction of 1.5% and 4.4%, respectively.

### Mechanistic counterfactual: contact duration by travel frequency

To examine whether the effect of removing lake contact duration varied across levels of travel frequency, we estimated conditional average treatment effects. Reductions in infection probability were minimal among non-travellers and infrequent visitors (≤once a month), highest among those travelling two times per month to two times per week (difference in infection risk of 6–10 percentage points—table 1). Daily travellers, while most at risk overall (figure 3), showed comparatively small reductions (3 percentage points reduction—table 1).

**Table 1:** Contact-only estimated change in infection probability under *do*(duration=0 minutes)

Travel frequency	Median Δ (risk difference)	95% crI (lower to upper)
Non-travellers	−0.030 (≈3%)	−0.071 to 0.002
Just once	−0.029 (≈3%)	−0.065 to 0.002
<Once per month	−0.034 (≈3%)	−0.079 to 0.003
Once per month	−0.038 (≈4%)	−0.088 to 0.003
Two times per month	−0.102 (≈10%)	−0.213 to 0.011
Once per week	−0.058 (≈6%)	−0.133 to 0.004
Two times per week	−0.082 (≈8%)	−0.158 to 0.008
Daily	−0.031 (≈3%)	−0.071 to 0.002

crI, credible interval.

### Sensitivity analysis

To assess robustness of our results, we conducted sensitivity analyses (see ‘Sensitivity analysis’ in online supplemental data). Our key findings for the probability of infection showed moderate to strong E-values for point estimates (travel effect=2.20, occupation effect=3.37) and weak to moderate for CIs, but these did not span zero (online supplemental table S12). Therefore, any unmeasured confounding variables were unlikely to fully explain the observed associations. Self-reported water contact duration was susceptible to underreporting bias; the direction of this bias is towards the null, meaning observed duration-infection estimates are conservative lower bounds of the true effect (online supplemental table S13). The travel-infection estimate was stable across all eight combinations of treating DAG mediators (MDA, activity type, duration) as potential confounders, with point estimates ranging from OR 1.49 to 1.74. Credible intervals remained entirely above null only when no mediators were conditioned on (model A, total effect) or when MDA alone was included (model B) (online supplemental table S14), confirming that MDA’s causal role does not influence the travel-infection conclusion. Models conditioning on activity type or duration crossed the null, consistent with the established partial mediation by these variables in the DAG (online supplemental table S14).

For the estimates drawn from counterfactual interventions in which daily travel was ceased, shared-water bounding scenarios which applied illustrative benefit-transfer fractions of 10%–30% to those that did not travel every day increased the estimated population-level reduction from 2.8 to between 3.7 and 5.6 percentage points, with credible intervals remaining entirely below zero across all scenarios. For behaviour substitution scenarios where it was assumed that a fraction (10%–50%) moved their water contact to other shared waterways, the benefit was reduced from 2.8 to between 2.5 and 1.4 percentage points (online supplemental table S15 and online supplemental figure S8).

## Discussion

This study aimed to test whether habitual movement of people living in low *S. mansoni* endemicity areas but travelling to high-transmission sites around Lake Victoria, Uganda, could be sustaining transmission in surrounding inland communities that have reached the WHO-defined threshold for EPHP.^2^ Our analysis revealed that frequent travel, particularly daily travel, is a plausible and quantifiable pathway for sustaining *S. mansoni* transmission in low-prevalence settings. Critically, our results indicate that multiple overlapping exposures contribute to infection. It is therefore unlikely that transmission can be controlled by a single intervention, highlighting the need for a systems-thinking approach that recognises behavioural, environmental, and structural factors influencing transmission dynamics in EPHP settings.

To explore how behavioural interventions might influence infection risk and assess potential resurgence pathways**,** we simulated a series of counterfactual scenarios using the fully resolved SCM. The aim was not to advocate for behavioural restrictions, but instead to represent idealised contrasts designed to quantify the causal contribution of specific behaviours to infection risk rather than prescribe interventions. We recognise that lake travel in this setting is often driven by economic and social livelihood and domestic necessity, making complete elimination unrealistic, but used the analysis to identify groups who might benefit most from enhanced surveillance or targeted treatment. Across all scenarios, predicted infection probabilities and mean EPG were lower than in the observed data. The largest reductions in infection were consistently observed within the specific subgroups targeted by each intervention, with relatively small effects at the population level, even when exploring a spillover effect. At the individual scale, such interventions could be considered successful, reducing infection probability by 12% in daily travellers, 26% in occupational visitors, and lowering mean EPG in those undertaking domestic water activities from 29 to 1. These changes would not only improve individual health outcomes but also reduce the likelihood of those individuals contributing to contamination of home-village water sources in the future. However, the insubstantial impact at the population level highlights a limitation of targeting individual risk factors to control transmission in established transmission settings. Building on these modelled insights, the empirical data show that daily travel to Lake Victoria was associated with a 1.7-fold higher probability of infection compared with no travel, confirming a causal link between travel frequency and infection. When potential mediators were included, this association became non-significant, indicating that the elevated risk among daily travellers is largely explained by lake-related activities and time spent in the water. Participants involved in occupational water contact had a 3.4-fold higher infection probability than those reporting no lake activity, spending on average 92 min in the lake compared with 14 min for domestic activities. Although domestic activities were not linked to higher infection probability, they were strongly associated with higher mean EPG among those infected, possibly reflecting occasional high-risk behaviours such as washing clothes (22% of domestic cases) in shallow shoreline waters where *Biomphalaria* spp (*B. sudanica*, *B. pfeifferi* and *B. choanomphala*) snails and cercarial densities are highest.^34^ Weekly travellers showed no elevated infection risk despite moderate exposure durations (~39–40 min), likely due to the low proportion engaged in occupational contact (4%).

Mechanistic counterfactual analysis, simulating zero minutes of lake contact while stratifying by travel frequency to assess the direct contribution of contact duration independent of mobility behaviour, revealed the greatest reductions in infection probability among those travelling two times per month, whereas daily travellers, the group at highest overall risk showed smaller changes, likely reflecting residual infection in this group. Taken together, these findings indicate that infection risk is shaped by the frequency, nature and duration of water contact, and that some transmission persists among individuals with little or no lake contact. This further supports the earlier evidence that multiple overlapping exposures contribute to infection risk. The detection of infections among participants who did not report having travelled to the lake in the last 3 months (28% in adults, 28% in SAC and 36% in PSAC), is consistent with local transmission foci being maintained by parasite importation from frequent travellers, rather than individuals needing to travel to the lake themselves to become infected. Frequent travellers therefore appear to play a pivotal role in seeding infections within their home communities through routine water contamination, allowing transmission to persist among non-travellers and across the wider area. This connectivity blurs the boundaries between ‘high-risk’ and ‘low-risk’ villages. Although all five study villages met EPHP criteria, with no heavy-intensity infections and low mean egg counts among egg-positive individuals (11 EPG, SD=20), these low-intensity but persistent infections have important public health implications, highlighting the risk of underestimating transmission potential and resurgence in post-EPHP settings, suggesting that reliance on egg counts alone underestimates the true reservoir.

We therefore suggest that control in settings which have achieved EPHP requires a systems-based approach that addresses more than just the proximal drivers of transmission (eg, human behaviour and environmental reservoirs) to acknowledge the multiplicative effects of distal drivers that are often socio-political and structural in nature.^35^ Our statistical framework provides a tool to assess the potential impact of such strategies without relying on large, highly parameterised dynamic models, which require extensive, context-specific data. By linking empirical behavioural data to infection outcomes, this approach can help understand mechanistic pathways which could lead to resurgence. Looking forward, this framework could also inform adaptive post-EPHP surveillance and intervention strategies, enabling programmes to anticipate and respond dynamically to early signals of re-emergence.

### Limitations and future work

This study relied on self-reported behaviour rather than direct observation, which was not feasible at this scale. Social desirability bias is a known limitation of self-reporting.^36,37^ Water contact duration carried the greatest misreporting risk given its continuous nature; if under-reporting occurred, our duration-infection estimates would be conservative lower bounds of the true effect, though this does not affect the primary travel estimate as duration is not included in the total effect model. MDA was susceptible to both over-reporting and systematic differences between participants and non-participants, such as in health-seeking behaviour, either of which could distort the MDA-infection relationship. Over-reporting in particular would dilute the apparent protective effect of MDA towards the null, meaning our observed null result may understate MDA’s true protective effect. Evidence of desirability bias was perhaps apparent in that 50% of PSAC reported participating in the last MDA despite not being eligible, with no paediatric formulation available in this region at the time of the study. Regardless of the source of uncertainty, the MDA-infection association was weak with credible intervals crossing the null, structural sensitivity analysis confirmed that treating MDA as a confounder rather than a mediator did not alter the primary travel-infection conclusion. The causal role of MDA therefore remains uncertain but does not affect the primary findings of this study.

We hypothesised that high levels of habitual travel could also increase the risk of missing MDA rounds, and our data supported this, with 28 individuals reporting they missed the last MDA due to travel. However, if missing MDA is possible, so is missing surveys such as our own study, and such individuals may be systematically under-represented in surveillance and/or research activities, and may bias both epidemiological estimates and intervention evaluations.^38^ In our setting, this implies that the infection risk and MDA non-participation associated with high travel frequency may be underestimated, precisely because the most mobile individuals may have been absent during data collection.

Due to the wide range of lake activities, responses were grouped into broad activity categories to allow sufficient sample sizes, this may have obscured finer distinctions in exposure intensity. Therefore, activity-specific risk within age groups could be explored in future work with larger samples.

The cross-sectional design adds some limitations. As data were collected at a single time point, seasonal climatic variation over time may have influenced travel and activity patterns, though as all five villages share the same geographic region any effects would be expected to operate consistently across communities, such that there would be no discernible differential effect between villages. This study design also means temporal ordering between travel and infection cannot be directly established. However, reverse causation is biologically implausible as water exposure is a necessary precondition for infection. If anything, morbidity may suppress travel in heavily infected individuals, introducing differential participation bias that would attenuate the travel-infection association towards null, suggesting our estimate is conservative. Unmeasured confounding remains a limitation of any observational study. Socioeconomic status is a plausible confounder in this setting, however, it was not measured directly, in part reflecting the well-documented difficulty of capturing socioeconomic status accurately in field settings of this kind.^39^ E-value analysis indicated moderate to high robustness of key findings against unmeasured confounding.

The DAG encodes structural assumptions about causal ordering that cannot be fully verified from observational data alone, and alternative causal structures cannot be excluded. In particular, if activity or duration are incorrectly treated as mediators rather than confounders, adjustment sets would differ and estimates could be biased in either direction. Sensitivity analysis across eight alternative adjustment sets showed that the direction and magnitude of the travel-infection association were preserved across all models, consistent with a robust underlying effect. What changed was statistical precision, conditioning on activity or duration widened the credible intervals such that they crossed the null, consistent with over-adjustment when true mediators are incorrectly treated as covariates. We consider the mediator interpretation biologically plausible because water contact activity and duration are direct consequences of travel to the lake, for example, a child who does not travel cannot engage in lake activity. We therefore interpret these findings as consistent with, rather than conclusive evidence of, the proposed mediation structure.

The environmental transmission potential of *S. mansoni* in the study villages themselves remains uncharacterised. Malacological surveys, cercarial density studies and eDNA-based water sampling have been conducted extensively along the Lake Victoria shoreline.^34,40^ However, equivalent environmental data do not exist for the inland communities that have achieved EPHP. This highlights the gap that our findings expose, if infection is being imported into inland communities by mobile individuals, the boundary between high-risk and low-risk zones may be epidemiologically meaningless, yet without environmental surveillance data from the inland villages themselves, the transmission potential of local water sources remains unknown. Future work combining malacological surveys, cercarial density estimates, and eDNA sampling in inland villages would allow direct testing of whether imported parasites are establishing local transmission cycles, and would provide the environmental evidence needed to move beyond the epidemiologically arbitrary boundary between high- and low-risk regions.

## Conclusions

The findings presented here suggest that, in regions which have achieved EPHP, a small subset of individuals engaging in high-risk behaviours may help to sustain local prevalence, particularly occupational lake contact and habitual travel, and may serve as a bridge in maintaining infection within otherwise low-risk groups. Counterfactual simulations showed that while targeted interventions substantially reduce risk in individuals, their effect at the population level is negligible, indicating that such approaches alone are insufficient. Preventing resurgence will therefore require a systems-based strategy that integrates human, environmental, structural, and ecological drivers of transmission to safeguard long-term gains and achieve durable interruption of *S. mansoni* transmission.

## Supplementary material

10.1136/bmjph-2025-004500 online supplemental file 1

## Data Availability

Data are available in a public, open access repository.
