# Supplementary material for: Local habitual movement as a mechanism for Schistosoma mansoni transmission resurgence: a causal analysis of cross-sectional survey data
Source: BMJ Public Health. 2026 Aug 14;4(3):e004500. doi: 10.1136/bmjph-2025-004500 (PMC13479501; doi:10.1136/bmjph-2025-004500)
Supplement: Supplementary file 1 [file bmjph-4-3-s001.docx]

# Local habitual movement as a mechanism for *Schistosoma mansoni* transmission resurgence – a causal analysis of cross-sectional survey data

Rivka M. Lim^1,2^*, Moses Arinaitwe^2^, Simon A. Babayan^2^, Andrina Nankasi^3^, Alon Atuhaire^3^, Annet Namukuta^3^, Mwima Nicholas ^3^, Amy B. Pedersen^1^, Joanne P. Webster^4^, Poppy H.L. Lamberton^2^•, Jessica Clark^2^•

^1^Institute of Evolution and Ecology, School of Biological Sciences, Ashworth Laboratories, University of Edinburgh, Edinburgh, UK

^2^School of Biodiversity, One Health and Veterinary Medicine, University of Glasgow, Glasgow, UK

^3^Vector Borne and Neglected Tropical Diseases Control Division, Ministry of Health, Kampala, Uganda

^4^Department of Pathobiology and Population Sciences, Royal Veterinary College, University of London, Herts, UK

### *corresponding authors: [rivka.lim@ed.ac.uk](mailto:rivka.lim@ed.ac.uk);

•joint senior authorship

**Supplementary Material**

## Village selection

To select villages for the main study a baseline survey was carried out in twenty villages which were all approximately five kilometres from Lake Victoria, Uganda. This distance was chosen as previous evidence suggested that villages over five kilometres from Lake Victoria were at significantly lower risk of infection with prevalence below 15% [1]. For baseline surveillance only the WHO guidelines for rapid assessment were followed; approximately fifty school-aged children (SAC) were sampled in each village [2]. Each child provided a single stool sample, which was examined using one Kato Katz thick smear slide [3]. Based on these initial surveys, five villages were selected for the main study which had all reached the WHO target of elimination of transmission which resulted in communities who had either low (<10%) or moderate (<50%) prevalence. Simulated power analyses were conducted during this process to ascertain the required sample sizes in each population <https://github.com/iamjessclark/travelStudySchisto>

In each village prior to study commencement a meeting was hosted by the Ministry of Health team to which all members of the community were invited. During this meeting the purpose and design of the study was explained and any questions answered.

**Table S1,** Outline of questions asked to participants during the interview on travel behaviour.

| **Variable** | **Description** | **Type** | **Options / Range** |
| --- | --- | --- | --- |
| Date | Date of questionnaire | Date |  |
| Location | Where questionnaire is being conducted | Character | District name |
| Name of landing site |  | Character |  |
| Unique ID | Identifier for each person. Even representation of men and women. Representative age sample. | Character | First three letters of district name_number allocated to person_1 or 2 for participant sex |
| Age | Age of the participant in whole number, not age group | Integer | 2 years+ |
| Gender | Gender of participant | Categorical | Male / Female |
| Occupation | Occupation of participant | Categorical | Businessperson, farmer, fisherman, fishmonger, homemaker, student, unemployed |
| Current place of residence | District/village where participant lives | Categorical | Please name village and district |
| Is that the same location as here? |  | Categorical | Yes / No |
| How long have you been in this place? | If not resident in survey location, how long are they staying for | Integer | Days / weeks / months |
| How long in current residence? | Duration of time in years participant has lived in said district | Categorical | <1 year, 1-2, 3-4, 5-6, 7-8, 9+ |
| In the last three months, how many times did you travel to [reservoir site]? | Three months is used because it is the time between data collection periods | Categorical | Daily, weekly, twice a month, once a month, less than once a month, just once |
| In the next three months, how many times will you travel to [reservoir site]? | To complement the last question and assess accuracy | Integer |  |
| Why do you make this journey? |  | Categorical | Write-in entry |
| Do you always complete these journeys? | Metric to understand whether the journey is always completed or sometimes abandoned | Categorical | Yes, no, sometimes |
| If not, how often do you abandon your journey? | Metric of journey completion necessary for model fitting | Categorical | At least once a day/week/month; 25%, 50%, 75% of the time |
| If not resident here, number of journeys to this location in the last 3 months | To understand broader migratory patterns | Integer |  |
| How has COVID impacted this? | To understand if this is a normal behaviour pattern | Categorical | I travel more often / less often / it has not changed |
| On average, how long do you stay? | Duration in days/weeks/months | Integer | Days / weeks / months |
| Are there latrines available for you to use when you are here? |  | Categorical | Yes / No |
| Do you use a latrine when you are here? |  | Categorical | Yes / No |
| Where do you defecate if there is no latrine when you are here? | To capture where people go to the toilet when the scenario is not optimal | Categorical | Write-in entry |
| Do you come into contact with water here? | Does the participant come into contact with water in the survey location? | Categorical | Yes / No |
| How long do you spend in the water here? |  | Integer | Minutes |
| How deeply submerged when you are here? | To understand the impact of water contact patterns | Categorical | Ankle, thighs, stomach, chest, fully submerged |
| Do you come into contact with water in [reservoir location]? | To determine water contact patterns | Categorical | Yes / No |
| How long do you spend in the water in [reservoir location]? |  | Integer | Minutes |
| How deeply submerged when you are in [reservoir site]? | To understand differences in water contact behaviour at home and when away | Categorical | Ankle, thighs, stomach, chest, fully submerged |
| Are there latrines available for you to use when you are in [reservoir location]? |  | Categorical | Yes / No |
| Do you use a latrine when you are in [reservoir location]? | To understand differences in behaviour between home and travelled-to locations | Categorical | Yes / No |
| Where do you defecate if there is no latrine available in [reservoir location]? |  | Categorical | Write-in entry |
| How do you get to [reservoir site]? |  | Categorical | Boda boda, matatu, friend's vehicle, private vehicle, bicycle, walk, boat |
| Is there anywhere else that you often travel to for water? |  | Categorical |  |
| Did you participate in the most recent schistosomiasis MDA here? | To understand MDA interactions | Categorical | Yes / No / Not sure |
| If no, why? |  | Categorical | Away for work; took part elsewhere; away for recreation; not infected; don't need medicine; COVID; not offered medicine; do not trust medicine; safety concerns (e.g. pregnant); other |
| Will you participate in the next MDA? |  | Categorical | Yes / No / Maybe |
| If no or maybe, what might make you participate? |  | Character | Write-in entry |
| If you participated elsewhere, where did you participate? |  | Character | Please list location |

**Table S2.** Participants responses to what activity they did at Lake Victoria put into groups for statistical analysis.

| **Activity response** | **Grouping** |
| --- | --- |
| Fetch water for home use | Domestic |
| Fetch water for work use |  |
| Water |  |
| Washing |  |
| Wash |  |
| Going to the garden |  |
| Cultivating |  |
| Cross bypassing |  |
| Boarding a boat |  |
| Go to work | Occupation |
| Fishing |  |
| Ferrying bricks |  |
| Swimming | Recreation |
| Recreation |  |
| Play |  |
| Motorcycle |  |
| Bathing |  |
| Visit relative | Trade/Visit |
| Visit friends |  |
| Trade |  |
| Sell jackfruits |  |
| To buy food |  |
| Selling ripes |  |
| Sell cassava |  |
| School |  |
| Prayers |  |
| Operating small drinking place |  |
| Market |  |
| Friends |  |
| For vegetables |  |
| Food |  |
| Fishmonger |  |
| Buying fish |  |
| Buying clothes |  |
| Buy silverfish |  |

Model validation and restructuring

The structure of the DAG was validated against the observed data by testing the implied conditional independencies between the variables. The implied independencies were as follows: age was independent of sex; duration independent of MDA if conditioned on age, location and travel or activity, age, location and sex; MDA independent from sex when conditioned on age, location and travel. To identify and test these implied independencies, we use the concept of d-separation where two variables are d-separated given a set of conditioning variables if all paths between them are "blocked". A path is blocked if it contains a collider (a node with two arrows pointing towards it, e.g. A → B ← C) that is not conditioned on (nor any of its descendants), or if it contains a fork (e.g. A ← B → C) that is conditioned on (Pearl et al., 2016). This testing was carried out by constructing a general linear model (GLM) in R, using the appropriate link function, with the outcome as the response variable and the exposure the predictor (corresponding to the direction of the assumed causal influence in the DAG). Additional variables were included in the model based on the conditioning rules outlined above. Independence between the variable and the outcome was assumed if the p-value between the outcome and the exposure variable from the GLM was >0.1.

If a conditional independence did not hold in the data, the DAG structure was updated to include additional plausible paths or modify existing ones. This process was repeated until the DAG was both biologically meaningful and all implied conditional independencies were consistent with the observed data. This final validated DAG was then used to identify appropriate adjustment sets for estimating both total and direct effects across the causal framework.

The final structure of the DAG was as follows: Age and sex were exogenous variables with no parent nodes, therefore influencing downstream behavioural and exposure related variables. Age and sex class were treated as temporally prior to location based on the assumption that demographic characteristics influence where individuals live [4]. Location was exogenous to all other variables to capture spatial heterogeneity in exposure risk. The relationship between travel frequency and infection had both indirect and direct effects. This relationship was indirectly mediated by activity type as those who travel more frequently are more likely to engage in regular activities like chores (domestic) or work (occupation) [5], and also mediated by duration, this was decided by causal priority [4], since individuals must first choose to travel to the lake before they enter it. A direct path from travel frequency to infection was also included to capture potential exposure mechanisms not fully explained by duration or activity, such as unmeasured behavioural factors. Activity type influenced duration, reflecting that certain activities involve longer exposure [5–7]. Duration in the lake was a direct exposure variable that causally influenced infection [8,9]. MDA history was assumed to influence infection, as treatment reduces worm burden [10]. Travel frequency was also assumed to have a negative effect on MDA as those who travel often may not be present at their home village when the MDA took place [11].

**Table S3.** Minimal adjustment sets used in total and direct effect models by exposure.

|  | **Minimal adjustment set** | |
| --- | --- | --- |
| **Exposure** | **Total effect** | **Direct effect** |
| Travel frequency | Age, Sex, Location | Age, Sex, Location, MDA and Activity |
| Activity | Age, Sex, Location and Travel frequency | Age, Sex, Location, Travel frequency and Duration |
| MDA | Age, Location and Travel frequency | Age, Location and Travel frequency |
| Duration | Age, Sex, Location and Activity | Age, Sex, Location and Activity |

Sensitivity analysis of priors for the coefficients

For all Bayesian analyses, we used weakly informative priors, as there was little to no prior data available to support more specific assumptions. To assess the sensitivity of our findings to prior choice, we also fitted models with narrower coefficient priors: Normal(0, 5) and Normal(0, 2) (Table S3). The most notable difference under these more informative priors was a reduction in the estimated effect of activity on egg burden (EPG) (Figure S1). However, the relative ranking and direction of effects across activity categories remained consistent, with domestic activity showing the strongest effect. This effect remained strongly supported, as its posterior distribution did not cross the null (Figure S1A). Under the more restrictive (0, 2) priors, although the pattern of relative effects remained unchanged, the increased regularisation introduced by the tighter prior led to greater uncertainty around the estimated effects, and all posterior distributions crossed the null, including that of domestic activity (Figure S1B).

This is likely due to the relatively small number of individuals with positive egg counts, which limits the information available to overcome prior shrinkage. Despite this, we believe that the use of weakly informative priors remains justified, given the exploratory nature of the analysis and the absence of prior knowledge in this setting.

Using narrower coefficient priors made no meaningful difference when estimating the effect of travel frequency, MDA nor duration in the lake.

**Figure S1.** Total effects of activity on *Schistosoma mansoni* infection burden.

Posterior estimated total effects of lake-related activities, on the log mean of *S. mansoni* eggs per gram of stool. The model included fixed effects for activity type (domestic, occupational, recreational, trade/visit), and was adjusted for age class, sex, and location and duration.

Posterior densities represent marginal distributions of estimated effects, with the vertical dashed line indicating the null (log = 0).

A) Moderately informative coefficient priors of Normal(0,5)
B) Conservative coefficient priors of Normal(0,2)

**Table S4.** Model parameters and prior distributions.

| **Model** | **Parameter** | **Prior** | **Notes** |
| --- | --- | --- | --- |
| Bernoulli (Infection status) | Intercept | Normal(1.5, 2.5) | Expected baseline infection probability of ~18% |
| Bernoulli (Infection status) | Regression coefficients | Normal(0, 10) | Weakly informative, no strong prior knowledge |
| Gamma (EPG) |  | Normal(2.5, 1) [log scale] | Approximates log mean of observed egg counts |
| Gamma (EPG) | Regression coefficients | Normal(0, 10) | Weakly informative, allows potentially large effects |
| Gamma (EPG) | Shape parameter | Exponential(1) | Allows for overdispersion |
| Sensitivity Analysis | Regression coefficients - moderate | Normal(0, 5) | Moderate prior, intercept and exponential as above |
| Sensitivity Analysis | Regression coefficients - conservative | Normal(0, 2) | Conservative prior, intercept and exponential as above |

**Table S5.** MCMC convergence diagnostics (R-hat and effective sample size) for Bayesian models of *S. mansoni* infection prevalence and egg burden

| **Model** | **Variable** | **mean** | **sd** | **q2.5** | **q97.5** | **rhat** | **ess_bulk** | **ess_tail** |
| --- | --- | --- | --- | --- | --- | --- | --- | --- |
| Travel -> Infection | β Intercept | -0.543 | 0.273 | -1.08 | -0.006 | 1.0001 | 23257 | 22105 |
| Travel -> Infection | β travel frequency1 | 0.018 | 0.36 | -0.7 | 0.72 | 1.0002 | 42895 | 22379 |
| Travel -> Infection | β travel frequency2 | -0.143 | 1.53 | -3.464 | 2.604 | 1.0002 | 47642 | 17681 |
| Travel -> Infection | β travel frequency3 | 0.576 | 0.336 | -0.079 | 1.234 | 1.0005 | 42526 | 22212 |
| Travel -> Infection | β travel frequency6 | 1.298 | 0.7 | -0.029 | 2.717 | 1 | 49478 | 21601 |
| Travel -> Infection | β travel frequency13 | -0.218 | 0.34 | -0.896 | 0.442 | 0.9999 | 37315 | 22919 |
| Travel -> Infection | β travel frequency26 | 0.762 | 0.482 | -0.179 | 1.71 | 1.0004 | 45188 | 22436 |
| Travel -> Infection | β travel frequency92 | 0.552 | 0.249 | 0.062 | 1.04 | 1 | 32193 | 24438 |
| Travel -> Infection | β age class PSAC | 0.022 | 0.249 | -0.463 | 0.513 | 1.0001 | 39047 | 23050 |
| Travel -> Infection | β age class SAC | 0.053 | 0.213 | -0.364 | 0.467 | 1 | 44538 | 23463 |
| Travel -> Infection | β sex | 0.22 | 0.19 | -0.153 | 0.594 | 1.0003 | 54016 | 19840 |
| Travel -> Infection | β LocationB | -0.679 | 0.29 | -1.246 | -0.109 | 1.0001 | 24995 | 23117 |
| Travel -> Infection | β LocationNam | -0.633 | 0.298 | -1.218 | -0.056 | 1.0001 | 26191 | 22808 |
| Travel -> Infection | β LocationNan | 0.262 | 0.297 | -0.32 | 0.846 | 1.0003 | 28392 | 23968 |
| Travel -> Infection | β LocationS | -0.708 | 0.291 | -1.287 | -0.142 | 1 | 24296 | 23007 |
| Travel -> Infection | Intercept | -0.618 | 0.09 | -0.796 | -0.442 | 1 | 52951 | 21566 |
| Activity -> Infection | β Intercept | -1.316 | 0.276 | -1.86 | -0.783 | 1.0002 | 21544 | 20709 |
| Activity -> Infection | β Domestic | -0.349 | 0.459 | -1.26 | 0.54 | 1.0001 | 10420 | 15929 |
| Activity -> Infection | β Occupation | 1.22 | 0.569 | 0.112 | 2.355 | 1.0001 | 14579 | 17689 |
| Activity -> Infection | β Recreation | 0.172 | 0.474 | -0.761 | 1.099 | 1.0001 | 12981 | 18839 |
| Activity -> Infection | β TradeORVisit | 0.159 | 0.383 | -0.586 | 0.909 | 1.0001 | 10662 | 15728 |
| Activity -> Infection | β age class PSAC | 0.093 | 0.256 | -0.406 | 0.596 | 1.0001 | 25159 | 20789 |
| Activity -> Infection | β age class SAC | 0.063 | 0.224 | -0.381 | 0.504 | 1.0001 | 26085 | 20627 |
| Activity -> Infection | β sex | 0.133 | 0.197 | -0.256 | 0.517 | 1.0003 | 27368 | 21427 |
| Activity -> Infection | β LocationB | 0.81 | 0.311 | 0.203 | 1.423 | 1 | 19279 | 20038 |
| Activity -> Infection | β LocationNam | 0.399 | 0.346 | -0.281 | 1.083 | 1.0003 | 18208 | 20452 |
| Activity -> Infection | β LocationNan | 0.965 | 0.32 | 0.342 | 1.588 | 1.0002 | 21238 | 21008 |
| Activity -> Infection | β LocationS | 0.026 | 0.303 | -0.568 | 0.622 | 1.0003 | 21073 | 20938 |
| Activity -> Infection | β travel_frequency1 | -0.102 | 0.443 | -0.98 | 0.757 | 1 | 14164 | 19310 |
| Activity -> Infection | β travel_frequency2 | -0.097 | 1.569 | -3.514 | 2.715 | 1.0002 | 23696 | 16030 |
| Activity -> Infection | β travel_frequency3 | 0.446 | 0.459 | -0.452 | 1.342 | 1.0001 | 12197 | 18032 |
| Activity -> Infection | β travel_frequency6 | 0.807 | 0.789 | -0.714 | 2.385 | 1 | 20173 | 19959 |
| Activity -> Infection | β travel frequency13 | -0.203 | 0.48 | -1.146 | 0.733 | 1.0002 | 12469 | 18073 |
| Activity -> Infection | β travel frequency26 | 0.65 | 0.569 | -0.461 | 1.766 | 1 | 15191 | 18573 |
| Activity -> Infection | β travel frequency92 | 0.541 | 0.401 | -0.24 | 1.333 | 1.0002 | 10261 | 15671 |
| Activity -> Infection | Intercept | -0.621 | 0.092 | -0.804 | -0.443 | 0.9999 | 29653 | 20367 |
| MDA -> Infection | β_Intercept | -0.36 | 0.291 | -0.935 | 0.21 | 1.0001 | 28000 | 22268 |
| MDA -> Infection | β MDA | -0.142 | 0.221 | -0.571 | 0.293 | 1 | 45693 | 23393 |
| MDA -> Infection | β travel_frequency1 | 0.023 | 0.36 | -0.698 | 0.717 | 0.9999 | 41871 | 22550 |
| MDA -> Infection | β travel_frequency2 | -0.191 | 1.528 | -3.555 | 2.537 | 1.0002 | 46524 | 16934 |
| MDA -> Infection | β travel_frequency3 | 0.579 | 0.337 | -0.086 | 1.245 | 1.0003 | 42383 | 23347 |
| MDA -> Infection | β travel_frequency6 | 1.293 | 0.701 | -0.04 | 2.725 | 1.0001 | 44065 | 20385 |
| MDA -> Infection | β travel frequency13 | -0.26 | 0.338 | -0.938 | 0.387 | 1.0001 | 35426 | 23948 |
| MDA -> Infection | β travel frequency26 | 0.8 | 0.475 | -0.133 | 1.745 | 1.0002 | 44818 | 21945 |
| MDA -> Infection | β travel frequency92 | 0.564 | 0.248 | 0.086 | 1.049 | 1.0001 | 31141 | 24055 |
| MDA -> Infection | β age class PSAC | 0.038 | 0.247 | -0.447 | 0.52 | 1.0002 | 39816 | 22357 |
| MDA -> Infection | β age class SAC | 0.069 | 0.212 | -0.346 | 0.481 | 1.0004 | 41875 | 23660 |
| MDA -> Infection | β LocationB | -0.666 | 0.296 | -1.244 | -0.089 | 1 | 23051 | 23585 |
| MDA -> Infection | β LocationNam | -0.656 | 0.296 | -1.245 | -0.084 | 1 | 24528 | 23824 |
| MDA -> Infection | β LocationNan | 0.277 | 0.298 | -0.307 | 0.859 | 1.0002 | 25460 | 23512 |
| MDA -> Infection | β LocationS | -0.722 | 0.293 | -1.306 | -0.157 | 1 | 22847 | 22664 |
| MDA -> Infection | Intercept | -0.617 | 0.091 | -0.796 | -0.441 | 1.0001 | 49048 | 21692 |
| Travel -> Burden | b_Intercept | 2.577 | 0.537 | 1.585 | 3.686 | 1.0003 | 16832 | 17531 |
| Travel -> Burden | β travel_frequency1 | 0.955 | 0.768 | -0.51 | 2.5 | 1.0002 | 15504 | 19122 |
| Travel -> Burden | β travel_frequency2 | -2.904 | 1.792 | -5.623 | 1.419 | 1 | 17369 | 13093 |
| Travel -> Burden | β travel_frequency3 | -0.273 | 0.8 | -1.735 | 1.429 | 1 | 17910 | 16641 |
| Travel -> Burden | β travel_frequency6 | -0.331 | 0.737 | -1.712 | 1.197 | 1.0001 | 16405 | 19186 |
| Travel -> Burden | β travel frequency13 | 0.327 | 0.675 | -0.942 | 1.734 | 1.0003 | 17183 | 18311 |
| Travel -> Burden | β travel frequency26 | -0.778 | 0.6 | -1.908 | 0.442 | 1.0001 | 18902 | 18688 |
| Travel -> Burden | β travel frequency92 | -0.042 | 0.5 | -1.044 | 0.919 | 1.0002 | 16045 | 17773 |
| Travel -> Burden | β age class PSAC | -0.258 | 0.623 | -1.454 | 1.007 | 1.0004 | 17909 | 19470 |
| Travel -> Burden | β age class SAC | -0.183 | 0.405 | -0.984 | 0.615 | 1.0002 | 22190 | 20797 |
| Travel -> Burden | β sex | 0.421 | 0.367 | -0.297 | 1.142 | 1.0002 | 20704 | 19581 |
| Travel -> Burden | β LocationB | -0.067 | 0.52 | -1.107 | 0.944 | 1.0001 | 15735 | 17799 |
| Travel -> Burden | β LocationNam | -2.424 | 1.123 | -4.309 | 0.088 | 1.0002 | 20488 | 15606 |
| Travel -> Burden | β LocationNan | -0.375 | 0.525 | -1.429 | 0.649 | 1 | 15894 | 18774 |
| Travel -> Burden | β LocationS | -0.359 | 0.655 | -1.638 | 0.959 | 1.0001 | 18841 | 20690 |
| Travel -> Burden | Intercept | 2.373 | 0.162 | 2.074 | 2.709 | 1.0002 | 19052 | 18915 |
| Activity -> Burden | b_Intercept | 2.436 | 0.537 | 1.442 | 3.569 | 1 | 23882 | 20529 |
| Activity -> Burden | β Domestic | 3.243 | 1.187 | 0.771 | 5.46 | 1.0002 | 7704 | 9419 |
| Activity -> Burden | β Occupation | 2.647 | 1.035 | 0.423 | 4.482 | 1.0003 | 7093 | 8722 |
| Activity -> Burden | β Recreation | 1.694 | 1.074 | -0.584 | 3.642 | 1.0003 | 7831 | 9496 |
| Activity -> Burden | β TradeORVisit | 2.117 | 1 | -0.045 | 3.893 | 1.0002 | 6895 | 8502 |
| Activity -> Burden | β age class PSAC | 0.009 | 0.594 | -1.101 | 1.241 | 1.0002 | 21280 | 19294 |
| Activity -> Burden | β age class SAC | 0.171 | 0.423 | -0.642 | 1.02 | 1 | 19814 | 19063 |
| Activity -> Burden | β sex | 0.436 | 0.379 | -0.32 | 1.173 | 1 | 23249 | 20337 |
| Activity -> Burden | β LocationB | -0.32 | 0.561 | -1.437 | 0.77 | 1 | 18379 | 18476 |
| Activity -> Burden | β LocationNam | -3.574 | 1.229 | -5.835 | -0.964 | 1 | 19150 | 16784 |
| Activity -> Burden | β LocationNan | -0.517 | 0.507 | -1.534 | 0.471 | 1.0001 | 18872 | 18764 |
| Activity -> Burden | β LocationS | -0.181 | 0.6 | -1.342 | 1.003 | 1.0002 | 20078 | 20207 |
| Activity -> Burden | β travel_frequency1 | -1.695 | 1.187 | -3.862 | 0.807 | 1.0002 | 7747 | 10400 |
| Activity -> Burden | β travel_frequency2 | -5.133 | 1.946 | -8.33 | -0.708 | 1.0001 | 11534 | 14136 |
| Activity -> Burden | β travel_frequency3 | -2.271 | 1.189 | -4.428 | 0.238 | 1.0002 | 7849 | 10206 |
| Activity -> Burden | β travel_frequency6 | -2.311 | 1.137 | -4.363 | 0.112 | 1.0002 | 8046 | 10589 |
| Activity -> Burden | β travel frequency13 | -2.046 | 1.124 | -4.093 | 0.31 | 1.0003 | 7121 | 9347 |
| Activity -> Burden | β travel frequency26 | -3.16 | 1.109 | -5.159 | -0.82 | 1.0002 | 7015 | 9161 |
| Activity -> Burden | β travel frequency92 | -2.044 | 0.957 | -3.711 | 0.053 | 1.0003 | 6691 | 8365 |
| Activity -> Burden | Intercept | 2.283 | 0.157 | 1.994 | 2.609 | 0.9999 | 18936 | 17780 |
| MDA-> Burden | b_Intercept | 2.697 | 0.7 | 1.349 | 4.127 | 1.0002 | 16498 | 16308 |
| MDA-> Burden | b_MDA | 0.096 | 0.523 | -0.957 | 1.092 | 1.0001 | 21401 | 18701 |
| MDA-> Burden | β travel_frequency1 | 0.657 | 0.777 | -0.82 | 2.236 | 1.0002 | 18058 | 19388 |
| MDA-> Burden | β travel_frequency2 | -3.32 | 1.786 | -5.947 | 1.022 | 1.0005 | 18622 | 12655 |
| MDA-> Burden | β travel_frequency3 | -0.499 | 0.771 | -1.888 | 1.168 | 1.0001 | 20271 | 16515 |
| MDA-> Burden | β travel_frequency6 | -0.356 | 0.768 | -1.786 | 1.229 | 1.0002 | 18213 | 18810 |
| MDA-> Burden | β travel frequency13 | 0.104 | 0.655 | -1.115 | 1.46 | 1.0004 | 19784 | 18027 |
| MDA-> Burden | β travel frequency26 | -0.818 | 0.612 | -1.959 | 0.431 | 1.0001 | 20961 | 19231 |
| MDA-> Burden | β travel frequency92 | -0.117 | 0.494 | -1.096 | 0.848 | 1.0002 | 17647 | 19267 |
| MDA-> Burden | β age class PSAC | -0.275 | 0.715 | -1.647 | 1.163 | 1.0002 | 19396 | 19847 |
| MDA-> Burden | β age class SAC | -0.141 | 0.418 | -0.945 | 0.693 | 1.0001 | 24038 | 20136 |
| MDA-> Burden | β LocationB | 0.13 | 0.525 | -0.906 | 1.152 | 1.0001 | 19333 | 19231 |
| MDA-> Burden | β LocationNam | -2.524 | 1.13 | -4.408 | 0.009 | 1.0001 | 21466 | 16759 |
| MDA-> Burden | β LocationNan | -0.192 | 0.509 | -1.209 | 0.795 | 1.0004 | 18454 | 19703 |
| MDA-> Burden | β LocationS | -0.235 | 0.679 | -1.546 | 1.128 | 1 | 20658 | 20454 |
| MDA-> Burden | Intercept | 2.39 | 0.162 | 2.091 | 2.727 | 1.0001 | 22024 | 20022 |
| Duration -> Infection | β_Intercept | -0.542 | 0.285 | -1.109 | 0.011 | 1.0001 | 17738 | 19974 |
| Duration -> Infection | β_DurMin | 0.005 | 0.003 | 0 | 0.011 | 1.0003 | 29378 | 21740 |
| Duration -> Infection | β Domestic | -0.167 | 0.294 | -0.747 | 0.408 | 1.0001 | 22097 | 23106 |
| Duration -> Infection | β Occupation | 1.208 | 0.501 | 0.249 | 2.218 | 1.0003 | 26575 | 21735 |
| Duration -> Infection | β Recreation | 0.262 | 0.372 | -0.477 | 0.985 | 1.0001 | 24384 | 21424 |
| Duration -> Infection | β TradeORVisit | 0.331 | 0.258 | -0.18 | 0.835 | 1 | 26982 | 22757 |
| Duration -> Infection | β age class PSAC | 0.011 | 0.248 | -0.473 | 0.499 | 1.0001 | 28726 | 22726 |
| Duration -> Infection | β age class SAC | 0.019 | 0.218 | -0.405 | 0.446 | 1.0001 | 31688 | 21819 |
| Duration -> Infection | β sex | 0.157 | 0.191 | -0.218 | 0.534 | 1.0004 | 36964 | 22081 |
| Duration -> Infection | β LocationB | -0.657 | 0.293 | -1.238 | -0.086 | 1.0001 | 19580 | 22084 |
| Duration -> Infection | β LocationNam | -0.175 | 0.31 | -0.784 | 0.432 | 1.0001 | 22746 | 22955 |
| Duration -> Infection | β LocationNan | 0.183 | 0.321 | -0.446 | 0.815 | 1.0001 | 19532 | 22279 |
| Duration -> Infection | β LocationS | -0.739 | 0.313 | -1.357 | -0.128 | 1.0003 | 17943 | 21960 |
| Duration -> Infection | Intercept | -0.605 | 0.091 | -0.784 | -0.429 | 1.0003 | 39075 | 23219 |

**Table S6.** *Schistosoma mansoni* prevalence and mean eggs per gram of stool (EPG) ordered by travel frequency to Lake Victoria, Uganda

| **Travel frequency** | **Age class** | **Number of participants** | **Infected• (%)** | **Mean EPG* (standard deviation)** |
| --- | --- | --- | --- | --- |
| None | Adult | 115 | 28% | 8.6 (10.5) |
| None | SAC | 65 | 28% | 45.0 () |
| None | PSAC | 76 | 36% | 12.8 (11.5) |
| Just once | Adult | 24 | 29% | 51.0 (81.9) |
| Just once | SAC | 18 | 33% | 4.4 (3.7) |
| Just once | PSAC | 8 | 25% | 0.5 () |
| Less than once per month | Adult | 1 | 0% | 0 |
| Less than once per month | SAC | 2 | 50% | 0.2 () |
| Once per month | Adult | 29 | 45% | 5.7 () |
| Once per month | SAC | 10 | 70% | 6.6 (2.2) |
| Once per month | PSAC | 10 | 40% | 5.0 () |
| Twice per month | Adult | 10 | 60% | 9.4 (5.1) |
| Twice per month | PSAC | 1 | 100% | 0.2 () |
| Once per week | Adult | 15 | 40% | 15.8 (11.1) |
| Once per week | SAC | 24 | 33% | 8.3 (10.0) |
| Once per week | PSAC | 17 | 24% | 13.7 () |
| Twice per week | Adult | 11 | 55% | 5.1 (5.5) |
| Twice per week | SAC | 10 | 50% | 4.4 (4.0) |
| Twice per week | PSAC | 1 | 100% | 0 |
| Daily | Adult | 61 | 44% | 12.5 (14.4) |
| Daily | SAC | 60 | 40% | 9.4 (13.9) |
| Daily | PSAC | 17 | 35% | 0 |

•using a positive Kato-Katz or a G-score ≥3, *excluding zeros and only using Kato-Katz

**Table S7.** Frequency of Lake Victoria travel and distribution of reported activities, stratified by age group, sex, and village.

|  |  |  |  |  | **Activity (percentage of all those that reported travel to the Lake in this population)** | | | | | | | |
| --- | --- | --- | --- | --- | --- | --- | --- | --- | --- | --- | --- | --- |
| **Characteristic** | **n** | **Total days travelled in last 3 months (Percentage of population)** | | **Average number of days** | **Domestic** | | **Occupation** | | **Recreation** | | **Trade/Visit** | |
| Adult | 266 | 150 | (56) | 42 | 57 | (38) | 21 | (14) | 7 | (5) | 59 | (40) |
| SAC | 189 | 122 | (65) | 50 | 42 | (34) | 7 | (6) | 32 | (26) | 35 | (29) |
| PSAC | 130 | 54 | (42) | 35 | 26 | (48) | 0 | (0) | 9 | (17) | 13 | (24) |
| Female | 364 | 203 | (56) | 43 | 85 | (42) | 9 | (4) | 20 | (10) | 80 | (40) |
| Male | 220 | 123 | (56) | 45 | 40 | (33) | 19 | (15) | 28 | (23) | 27 | (21) |
| B | 119 | 75 | (63) | 35 | 37 | (49) | 8 | (11) | 24 | (32) | 6 | (8) |
| Nw | 133 | 77 | (58) | 46 | 8 | (10) | 8 | (10) | 18 | (23) | 29 | (38) |
| Nam | 111 | 79 | (71) | 68 | 71 | (90) | 1 | (1) | 1 | (1) | 6 | (8) |
| Nan | 91 | 59 | (65) | 23 | 6 | (10) | 8 | (14) | 4 | (7) | 39 | (66) |
| S | 131 | 39 | (56) | 35 | 3 | (8) | 3 | (8) | 1 | (3) | 27 | (69) |
| **Total** | **585** | **326** | **(56)** | **43** | **125** | **(38)** | **28** | **(9)** | **48** | **(15)** | **107** | **(33)** |
| *PSAC: preschool age children, SAC: school age children* | | | | | | | | | | | | |

**Table S8.** Activity type among age classes by schistosomiasis infection status

| **Age class** | **Activity** | **Sample size**  **(ss)** | **Infected**  **(n)** | **Prevalence**  **(% - n/ss)** | **Prevalence within age class**  **(% - n/n in age class)** |
| --- | --- | --- | --- | --- | --- |
| Adult | None | 106 | 30 | 28.3 | 30.9 |
| Adult | TradeORVisit | 71 | 28 | 39.4 | 28.9 |
| Adult | Domestic | 60 | 20 | 33.3 | 20.6 |
| Adult | Occupation | 22 | 15 | 68.2 | 15.5 |
| Adult | Recreation | 7 | 4 | 57.1 | 4.1 |
| PSAC | None | 79 | 26 | 32.9 | 57.8 |
| PSAC | Domestic | 27 | 8 | 29.6 | 17.8 |
| PSAC | TradeORVisit | 15 | 7 | 46.7 | 15.6 |
| PSAC | Recreation | 9 | 4 | 44.4 | 8.9 |
| SAC | None | 68 | 19 | 27.9 | 27.5 |
| SAC | Domestic | 43 | 15 | 34.9 | 21.7 |
| SAC | TradeORVisit | 37 | 16 | 43.2 | 23.2 |
| SAC | Recreation | 34 | 14 | 41.2 | 20.3 |
| SAC | Occupation | 7 | 5 | 71.4 | 7.2 |

**Figure S2.** Prevalence of *S. mansoni* by village and Sex. Prevalence measured by a positive Kato Katz from three duplicate slides or if a POC-CCA test had a G-score ≥3. F = female and M = male.

### ****Figure S3. Observed infection prevalence and intensity by behavioural and exposure variables.****

### Observed infection prevalence (top and middle rows) and infection intensity (bottom row) plotted against behavioural and exposure variables included in the structural causal model. Bars and ribbons show observed mean prevalence with 95% binomial confidence intervals. Points represent individual infections (1 = infected, 0 = uninfected), shown with jitter for visibility.

**(A)** Infection prevalence by travel frequency to Lake Victoria (visits in the past three months).
**(B)** Infection prevalence by participation in the most recent mass drug administration (MDA).
**(C)** Infection prevalence by activity type at Lake Victoria.
**(D)** Infection prevalence by time spent in the lake (binned durations).
**(E)** Infection intensity (EPG, log₁₊ scale) by time spent in the lake (continuous).
**(F–H)** Infection intensity (EPG, log₁₊ scale) by travel frequency (F), MDA participation (G), and activity type (H).

**Figure S4.** Direct effect of travel frequency on log odds of infection with *Schistosoma mansoni.*

Posterior distributions for the estimated direct effects of self-reported travel frequency to Lake Victoria on the probability of infection, relative to individuals reporting no travel in the preceding three months (reference category). Estimates are log-odds coefficients from a Bayesian logistic regression model with a Bernoulli likelihood and logit link function. The model included fixed effects for travel frequency, age class, sex (binary), and location and the mediator’s activity and MDA. Densities represent marginal posterior distributions for each travel frequency level, with the vertical dashed line indicating the null effect (log-odds = 0). Travel frequency levels are ordered from highest (92 days) to lowest (1 day) reported visits to the lake.

**Figure S5.** Direct effect of travel frequency on log mean on burden of *Schistosoma mansoni* eggs per gram in stool.

Posterior distributions for the estimated direct effects of self-reported travel frequency to Lake Victoria on mean eggs per gram of stool, relative to individuals reporting no travel in the preceding three months (reference category). Estimates are log mean coefficients from a Bayesian logistic regression model with a gamma likelihood and log link function. Densities represent marginal posterior distributions for each travel frequency level, with the vertical dashed line indicating the null effect (log-odds = 0). Travel frequency levels are ordered from highest (92 days) to lowest (1 day) reported visits to the lake.

**Figure S6.** Direct effects of activity on *Schistosoma mansoni* infection probability.

Posterior distributions for the estimated direct effects of lake-related activities, the log odds of infection. The model included fixed effects for activity type (domestic, occupational, recreational, trade/visit), and was adjusted for age class, sex, and location and duration.

Posterior densities represent marginal distributions of estimated effects, with the vertical dashed line indicating the null (log = 0).

**Figure S7.** Direct effects of activity on *Schistosoma mansoni* infection burden.

Posterior distributions for the estimated direct effects of lake-related activities, on the log mean of *S. mansoni* eggs per gram of stool. The model included fixed effects for activity type (domestic, occupational, recreational, trade/visit), and was adjusted for age class, sex, and location and duration.

Posterior densities represent marginal distributions of estimated effects, with the vertical dashed line indicating the null (log = 0).

**Table S9.** Self-reported reasons for not taking part in the last mass drug administration.

| **Reason for not taking part** | **Number of people** |
| --- | --- |
| Too young | 42 |
| Away | 28 |
| Did not know about it | 22 |
| Not offered it | 15 |
| Did not trust it / concerned about side effects | 13 |
| COVID affected decision | 8 |
| Not infected, so did not need it | 3 |
| Did not know why | 19 |

**Table S10.** Self-reported participation in the last MDA by age group.

| **Age group** | **Total population size** | **% who reported taking part in last MDA** |
| --- | --- | --- |
| PSAC | 130 | 50% |
| SAC | 189 | 82% |
| Adults | 266 | 77% |

**Table S11.** Frequency of Lake Victoria travel and distribution of reported activities, stratified by duration in the lake.

| **Travel frequency** | **Activity** | **n** | **Proportion of those in travel frequency group** | **Average duration in the lake – minutes (sd)** |
| --- | --- | --- | --- | --- |
| Never | None | 232 | 91% | 0 () |
|  | Domestic | 5 | 2% | 0 (0) |
|  | Occupation | 1 | 0% | 2 () |
|  | Recreation | 2 | 1% | 40 (28) |
|  | TradeORVisit | 16 | 6% | 7 (22) |
| Just once | None | 12 | 24% | 1 (3) |
|  | Domestic | 6 | 12% | 6 (7) |
|  | Occupation | 4 | 8% | 24 (27) |
|  | Recreation | 12 | 24% | 25 (25) |
|  | TradeORVisit | 16 | 32% | 18 (53) |
| Less than once a month | Domestic | 1 | 33% | 0 () |
|  | TradeORVisit | 2 | 67% | 30 (0) |
| Once a month | None | 1 | 2% | 20 () |
|  | Domestic | 13 | 27% | 8 (18) |
|  | Occupation | 6 | 12% | 45 (23) |
|  | Recreation | 2 | 4% | 40 (28) |
|  | TradeORVisit | 27 | 55% | 20 (34) |
| Twice a month | Occupation | 4 | 36% | 215 (237) |
|  | TradeORVisit | 7 | 64% | 1 (4) |
| Once a week | None | 1 | 2% | 5 () |
|  | Domestic | 28 | 50% | 39 (49) |
|  | Occupation | 2 | 4% | 38 (11) |
|  | Recreation | 9 | 16% | 40 (57) |
|  | TradeORVisit | 16 | 29% | 40 (37) |
| Twice a week | None | 2 | 9% | 1 (1) |
|  | Domestic | 4 | 18% | 45 (90) |
|  | Occupation | 3 | 14% | 260 (399) |
|  | Recreation | 5 | 23% | 52 (18) |
|  | TradeORVisit | 8 | 36% | 22 (28) |
| Daily | None | 5 | 4% | 5 (8) |
|  | Domestic | 73 | 53% | 5 (12) |
|  | Occupation | 9 | 7% | 64 (70) |
|  | Recreation | 20 | 14% | 29 (23) |
|  | TradeORVisit | 31 | 22% | 17 (24) |

**Sensitivity analysis**

E-value Sensitivity Analysis for Unmeasured Confounding

E-values quantify the minimum strength of association that an unmeasured confounder would need to have with both the exposure and the outcome simultaneously, on the risk ratio scale, to fully explain away an observed association (VanderWeele & Ding 2017). For binary outcomes modelled with logistic regression, the posterior median odds ratio and 95% credible interval bounds were converted to approximate risk ratios using the observed baseline infection risk in the reference group: RR = OR / ((1 − P₀) + P₀ × OR), where P₀ is the baseline risk. For burden outcomes (Gamma log-link), mean ratios were used directly.

The E-value was calculated as: E = RR + √(RR × (RR − 1)); for protective effects (RR < 1): E = 1/RR + √((1/RR) × (1/RR − 1)).

E-values were calculated for both the point estimate and the credible interval bound closest to the null (inner CrI E-value). When the CrI includes the null, the inner CrI E-value is 1 by definition. Interpretation thresholds (VanderWeele & Ding 2017): <1.25 very weak; 1.25–2.0 weak/moderate; 2.0–5.0 moderate/strong; >5.0 strong evidence against confounding explaining the result.

**Table S12.** E-value sensitivity analysis for the effects of lake contact exposures and MDA participation on *S. mansoni* infection prevalence and egg burden

| **Exposure** | **Outcome** | **Effect type** | **Effect est** | **Cri lo** | **Cri hi** | **RR or MR approx** | **Evalue estimate** | **Evalue inner cri** |
| --- | --- | --- | --- | --- | --- | --- | --- | --- |
| Daily travel vs. never | Infection (prevalence) | OR | 1.74 | 1.06 | 2.83 | 1.42 | 2.2 | 1.26 |
| Domestic vs. no lake activity | Infection (prevalence) | OR | 0.71 | 0.28 | 1.72 | 0.77 | 1.91 | 1 |
| Occupation vs. no lake activity | Infection (prevalence) | OR | 3.37 | 1.12 | 10.54 | 1.98 | 3.37 | 1.38 |
| Recreation vs. no lake activity | Infection (prevalence) | OR | 1.19 | 0.47 | 3 | 1.12 | 1.5 | 1 |
| TradeORVisit vs. no lake activity | Infection (prevalence) | OR | 1.17 | 0.56 | 2.48 | 1.12 | 1.48 | 1 |
| MDA participation vs. none | Infection (prevalence) | OR | 0.87 | 0.57 | 1.34 | 0.91 | 1.41 | 1 |
| Daily travel vs. never | Burden (EPG, infected only) | MR | 0.96 | 0.35 | 2.51 | 0.96 | 1.23 | 1 |
| Domestic vs. no lake activity | Burden (EPG, infected only) | MR | 26.95 | 2.16 | 235.13 | 26.95 | 53.39 | 3.75 |
| Occupation vs. no lake activity | Burden (EPG, infected only) | MR | 15.1 | 1.53 | 88.38 | 15.1 | 29.69 | 2.42 |
| Recreation vs. no lake activity | Burden (EPG, infected only) | MR | 5.8 | 0.56 | 38.15 | 5.8 | 11.08 | 1 |
| TradeORVisit vs. no lake activity | Burden (EPG, infected only) | MR | 8.93 | 0.96 | 49.07 | 8.93 | 17.34 | 1 |
| MDA participation vs. none | Burden (EPG, infected only) | MR | 1.11 | 0.38 | 2.98 | 1.11 | 1.46 | 1 |

*OR = odds ratio; RR = relative risk; MR = mean ratio; est = estimate; Cri = credible interval; EPG = eggs per gram*.

Measurement error bounding

Water contact duration is self-reported and susceptible to social desirability bias: participants who spend longer at the lake are likely to underreport their duration to please researchers. This implies proportional underreporting: observed duration = λ × true duration, where λ is the reporting fraction (e.g. 0.7 = participants report 70% of true duration). Rearranging: β_true = β_observed / λ. We applied this bounding correction to the duration coefficient from a Bernoulli logistic model (adjustment set: Activity, Age, Sex, Location) across reporting fractions λ = {0.5, 0.6, 0.7, 0.8, 0.9, 1.0}, representing 0-50% underreporting. Corrected cumulative ORs are reported at 30, 60, and 120 minutes of water contact, reflecting the distribution of minutues in the data (75th percentile = 5 min, 90th percentile = 60 min, maximum = 720 min). The direction of bias is toward null under the assumption that underreporting is non-differential with respect to infection status, meaning infected and uninfected participants underreport equally which is plausible given that infection status is unknown to participants at the time of interview. Importantly, because Duration is a mediator on the Travel → Activity → Duration → Infection pathway, measurement error in Duration does not affect the primary causal estimate: the total effect of travel on infection is estimated from a model that does not condition on Duration, and is therefore unaffected by this bias.

**Table S13.** Sensitivity analysis for self-reported water contact duration: odds ratios under varying degrees of underreporting

| Reporting fraction | Pct underreporting | OR at 30min | OR at 60min | OR at 120min |
| --- | --- | --- | --- | --- |
| 0.5 | 50% | 1.34 | 1.82 | 3.24 |
| 0.6 | 40% | 1.28 | 1.63 | 2.66 |
| 0.7 | 30% | 1.23 | 1.52 | 2.31 |
| 0.8 | 20% | 1.2 | 1.44 | 2.08 |
| 0.9 | 10% | 1.18 | 1.39 | 1.92 |
| 1 | 0% | 1.16 | 1.34 | 1.80 |

*Pct=percent; OR=odds ratio, min = minutes.*

Mediators as confounding variables

In the DAG, MDA participation, activity type, and water contact duration are mediators on the Travel to Infection pathway. The total effect model conditions only on the minimal adjustment set {Age, Sex, Location}. If any mediator is instead a confounder (driven by unmeasured common causes of both travel and infection), it should be included in the adjustment set. We tested all 2³ = 8 combinations of treating each mediator as a confounder (models A–H), fitting Bernoulli logistic models with wide priors (Normal(0,10) for coefficients, Normal(−1.5, 2.5) for intercept). Models were compared on point estimate stability and whether the 95% CrI cleared the null.

**Table S14.** DAG structural sensitivity analysis: effect of daily lake travel on *S. mansoni* infection prevalence under alternative adjustment sets, varying assumptions about the mediator roles of water contact activity, duration, and MDA participation

| Model | Mediators | Log OR med | Log OR lo | Log OR hi | OR med | OR lo | OR hi | Cri clears null |
| --- | --- | --- | --- | --- | --- | --- | --- | --- |
| A: Total effect (correct DAG) | None | 0.553 | 0.062 | 1.040 | 1.738 | 1.064 | 2.829 | TRUE |
| B: + MDA | MDA | 0.552 | 0.069 | 1.043 | 1.736 | 1.071 | 2.838 | TRUE |
| C: + Activity | Activity | 0.547 | -0.236 | 1.343 | 1.728 | 0.790 | 3.831 | FALSE |
| D: + Duration | Duration | 0.396 | -0.110 | 0.906 | 1.486 | 0.896 | 2.474 | FALSE |
| E: + Activity + MDA | Activity + MDA | 0.555 | -0.235 | 1.349 | 1.742 | 0.791 | 3.852 | FALSE |
| F: + MDA + Duration | MDA + Duration | 0.403 | -0.106 | 0.916 | 1.496 | 0.900 | 2.501 | FALSE |
| G: + Activity + Duration | Activity + Duration | 0.502 | -0.289 | 1.312 | 1.652 | 0.749 | 3.715 | FALSE |
| H: + Activity + MDA + Duration | All mediators | 0.515 | -0.274 | 1.302 | 1.674 | 0.761 | 3.678 | FALSE |

*OR = odds ratio; med = median; cri = credible interval*

Spillover and substitution bounding

Counterfactual simulations used predictions of the posterior distribution to generate individual-level predicted infection probabilities under observed and counterfactual (daily travel set to zero) conditions across all 28,000 posterior draws. Population prevalence difference = mean(counterfactual) − mean(observed) per posterior draw, summarised as median and 95% CrI. For spillover, the per-draw mean risk reduction among daily travellers was applied at fractions of 10–30% to non-daily-travellers (representing reduced environmental cercarial contamination in shared waterways). For behavioural substitution, the base population-level difference was scaled by (1 − substitution fraction), representing the proportion of daily travellers who merely displace water contact to alternative sites.

**Table S15.** Shared-water bounding analysis for environmental benefit transfer and behavioural substitution effects on the estimated difference in *S. mansoni* infection prevalence between daily travellers and non-daily-travellers. Benefit-transfer fractions (0-30%) and substitution fractions (10-50%) are illustrative bounds, not quantities estimated from the data. Benefit-transfer scenarios are optimistic (the estimated population-level reduction increases as non-daily-travellers benefit from reduced shared-water cercarial contamination) and substitution scenarios are pessimistic (the reduction diminishes as travellers displace contact to alternative sites); together they bracket the baseline estimate.

| Spillover fraction | Scenario | Diff med pct | Diff lo pct | Diff hi pct |
| --- | --- | --- | --- | --- |
| 0 | 0% shared water benefit to non-daily-travellers | -2.813 | -5.225 | -0.323 |
| 0.1 | 10% shared water benefit to non-daily-travellers | -3.724 | -6.918 | -0.427 |
| 0.2 | 20% shared water benefit to non-daily-travellers | -4.636 | -8.610 | -0.532 |
| 0.3 | 30% shared water benefit to non-daily-travellers | -5.547 | -10.302 | -0.636 |
| 0 | 0% behavioural substitution | -2.813 | -5.225 | -0.323 |
| 0.1 | 10% behavioural substitution | -2.532 | -4.703 | -0.290 |
| 0.2 | 20% behavioural substitution | -2.251 | -4.180 | -0.258 |
| 0.3 | 30% behavioural substitution | -1.969 | -3.658 | -0.226 |
| 0.5 | 50% behavioural substitution | -1.407 | -2.613 | -0.161 |

*Diff med pct = difference in median population prevalence percentage*

**Figure S8.** **Bounding analysis for the effect of eliminating daily lake travel on population-level *S. mansoni* infection prevalence under two violation scenarios.**

The y-axis shows the counterfactual minus observed population prevalence difference (%), where values below zero indicate a reduction in prevalence if daily travel were eliminated. The x-axis shows the assumed fraction of violation under each scenario. Teal: spillover benefit to non-daily-travellers, where a proportion of the treatment effect is assumed to leak to these individuals, amplifying the apparent benefit of eliminating daily travel. Pink: behavioural substitution, where a proportion of travellers are assumed to shift to alternative lake contact behaviours rather than ceasing exposure entirely, attenuating the estimated effect. Shaded ribbons show 95% credible intervals. The dashed horizontal line marks zero difference. The estimated prevalence reduction remains negative (beneficial) across all scenarios examined, suggesting the counterfactual conclusion is robust to plausible violations of the independent manipulation assumption.

**Supplementary References**

1	Kabatereine NB, Brooker S, Tukahebwa EM, *et al.* Epidemiology and geography of Schistosoma mansoni in Uganda: implications for planning control. *Trop Med Int Health*. 2004;9:372–80. doi: 10.1046/j.1365-3156.2003.01176.x

2	Crompton DWT, World Health Organization. Preventive chemotherapy in human helminthiasis : coordinated use of anthelminthic drugs in control interventions : a manual for health professionals and programme managers. *Chim Helminthiases Chez Homme Util Coord Médicam Anthelminthiques Pour Interv Lutte Man À Intent Prof Santé Adm Programme*. 2006;62.

3	Katz N, Chaves A, Pellegrino J. A simple device for quantitative stool thick-smear technique in Schistosomiasis mansoni. *Rev Inst Med Trop São Paulo*. 1972;14:397–400.

4	Ferguson KD, McCann M, Katikireddi SV, *et al.* Evidence synthesis for constructing directed acyclic graphs (ESC-DAGs): a novel and systematic method for building directed acyclic graphs. *Int J Epidemiol*. 2020;49:322–9. doi: 10.1093/ije/dyz150

5	Reitzug F, Kabatereine NB, Byaruhanga AM, *et al.* Current water contact and Schistosoma mansoni infection have distinct determinants: a data-driven population-based study in rural Uganda. *Nat Commun*. 2024;15:9530. doi: 10.1038/s41467-024-53519-4

6	Trienekens SCM, Faust CL, Meginnis K, *et al.* Impacts of host gender on Schistosoma mansoni risk in rural Uganda-A mixedmethods approach. *PLoS Negl Trop Dis*. 2020;14:1–19.

7	Sow S, de Vlas SJ, Stelma F, *et al.* The contribution of water contact behavior to the high Schistosoma mansoni Infection rates observed in the Senegal River Basin. *BMC Infect Dis*. 2011;11:198. doi: 10.1186/1471-2334-11-198

8	Arinaitwe M, Adriko M, Kibwika B, *et al.* Residence time, water contact, and age-driven Schistosoma mansoni infection in hotspot communities in Uganda. *Am J Trop Med Hyg*. 2021;105:1772–81. doi: 10.4269/ajtmh.21-0391

9	Reitzug F, Ledien J, Chami GF. Associations of water contact frequency, duration, and activities with schistosome infection risk: A systematic review and meta-analysis. *PLoS Negl Trop Dis*. 2023;17:e0011377. doi: 10.1371/journal.pntd.0011377

10	Exum NG, Kibira SPS, Ssenyonga R, *et al.* The prevalence of schistosomiasis in Uganda: A nationally representative population estimate to inform control programs and water and sanitation interventions. *PLoS Negl Trop Dis*. 2019;13:e0007617. doi: 10.1371/journal.pntd.0007617

11	Adriko M, Faust CL, Carruthers LV, *et al.* Low praziquantel treatment coverage for Schistosoma mansoni in Mayuge district, Uganda, due to the absence of treatment opportunities, rather than systematic non-compliance. *Trop Med Infect Dis*. 2018;3:111.
